# Supplementary material for: Bidirectional communication between nucleotide and substrate binding sites in a type IV multidrug ABC transporter
Source: Nat Commun. 2025 Nov 11;16:9921. doi: 10.1038/s41467-025-65037-y (PMC12606247; doi:10.1038/s41467-025-65037-y)
Supplement: Supplementary file 1 — Supplementary Information [file 41467_2025_65037_MOESM1_ESM.docx]

**Supporting Information for**

**Bidirectional communication between nucleotide and substrate binding sites in a type IV multidrug ABC transporter**

Victor Hugo Pérez Carrillo^1,^*, Margot Di Cesare^2,^*, Dania Rose-Sperling^1^, Waqas Javed^2^ , Hannes Neuweiler^3^, Julien Marcoux^4,5^, Cédric Orelle^2,#^, Jean-Michel Jault^2,#^ and Ute A. Hellmich^1, 6, 7, #^

^1^Faculty of Chemistry and Earth Sciences, Institute of Organic Chemistry and Macromolecular Chemistry, Friedrich Schiller University Jena, Humboldtstraße 10, 07743, Jena, Germany

^2^ Molecular Microbiology and Structural Biochemistry (MMSB), UMR 5086 CNRS/University of Lyon, Lyon, France

^3^Department of Biotechnology & Biophysics, Julius-Maximilians-University Würzburg, Am Hubland, 97074 Würzburg, Germany

^4^Institut de Pharmacologie et de Biologie Structurale (IPBS), Université de Toulouse, CNRS, Université de Toulouse, Toulouse 31077, France

^5^Infrastructure Nationale de Protéomique, ProFI, UAR 2048, Toulouse, France

^6^Cluster of Excellence “Balance of the Microverse”, Friedrich Schiller University Jena, 07743, Jena, Germany

^7^Centre for Biomolecular Magnetic Resonance (BMRZ), Goethe University, Max von Laue Str. 9, 60438, Frankfurt, Germany

* These authors contributed equally

^#^ These authors jointly supervised this work: [cedric.orelle@cnrs.fr](mailto:cedric.orelle@cnrs.fr); [jean-michel.jault@cnrs.fr](mailto:jean-michel.jault@cnrs.fr); [ute.hellmich@uni-jena.de](mailto:ute.hellmich@uni-jena.de)


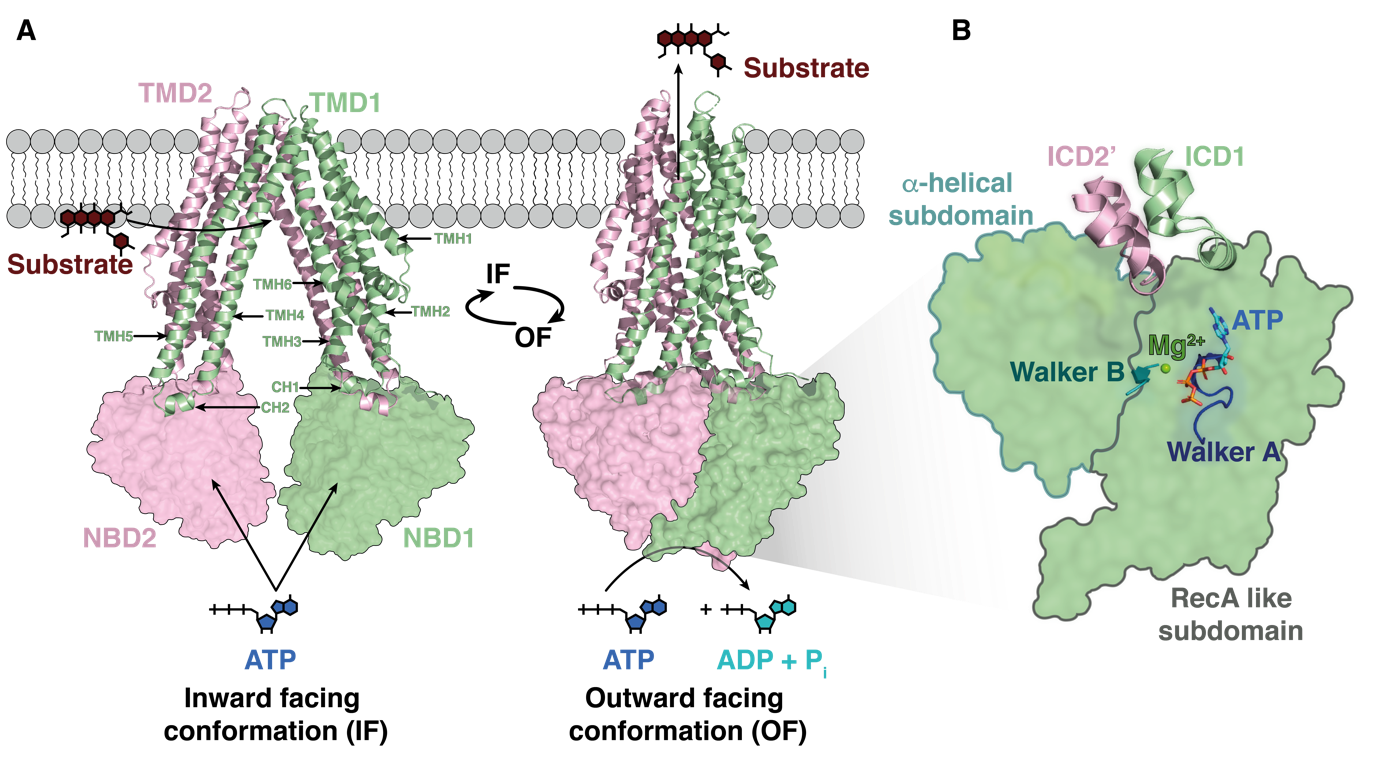


**Supplementary Figure 1: General architecture and mechanism of transport in type IV ABC transporters.** (**A**) Type IV ABC transporters are composed of two transmembrane domains (TMDs, shown as ribbons) and two nucleotide binding domains (NBDs, shown as surfaces). Each TMD is composed of six transmembrane helices (TMH). Type IV ABC transporters feature a ‘swapped domain’ topology, with each intracellular domain (ICD) of the TMD interacting with both the NBD of its own (in cis), as well as of the opposing subunit (in trans) via two coupling helices, CH1 and CH2. In general, type IV ABC transporters follow an alternative access mechanism. Substrate and nucleotide binding to the transporter in an inward-facing conformation (IF, PDB ID: 8QOE^1^) results in conformational changes that lead to an outward facing (OF, PDB ID: 6R81^2^) conformation. Upon ATP hydrolysis and release of substrate, the transporter can cycle back to the inward facing conformation (IF). (**B**) The NBDs are composed of two subdomains, the Rec A like domain and the α-helical subdomain. The Walker A and B motifs are necessary for binding and hydrolysis of ATP. The coupling helices, CH1 and CH2, from the intracellular domains (ICDs) at the TMD–NBD interface play a key role for long-range signal transmission.


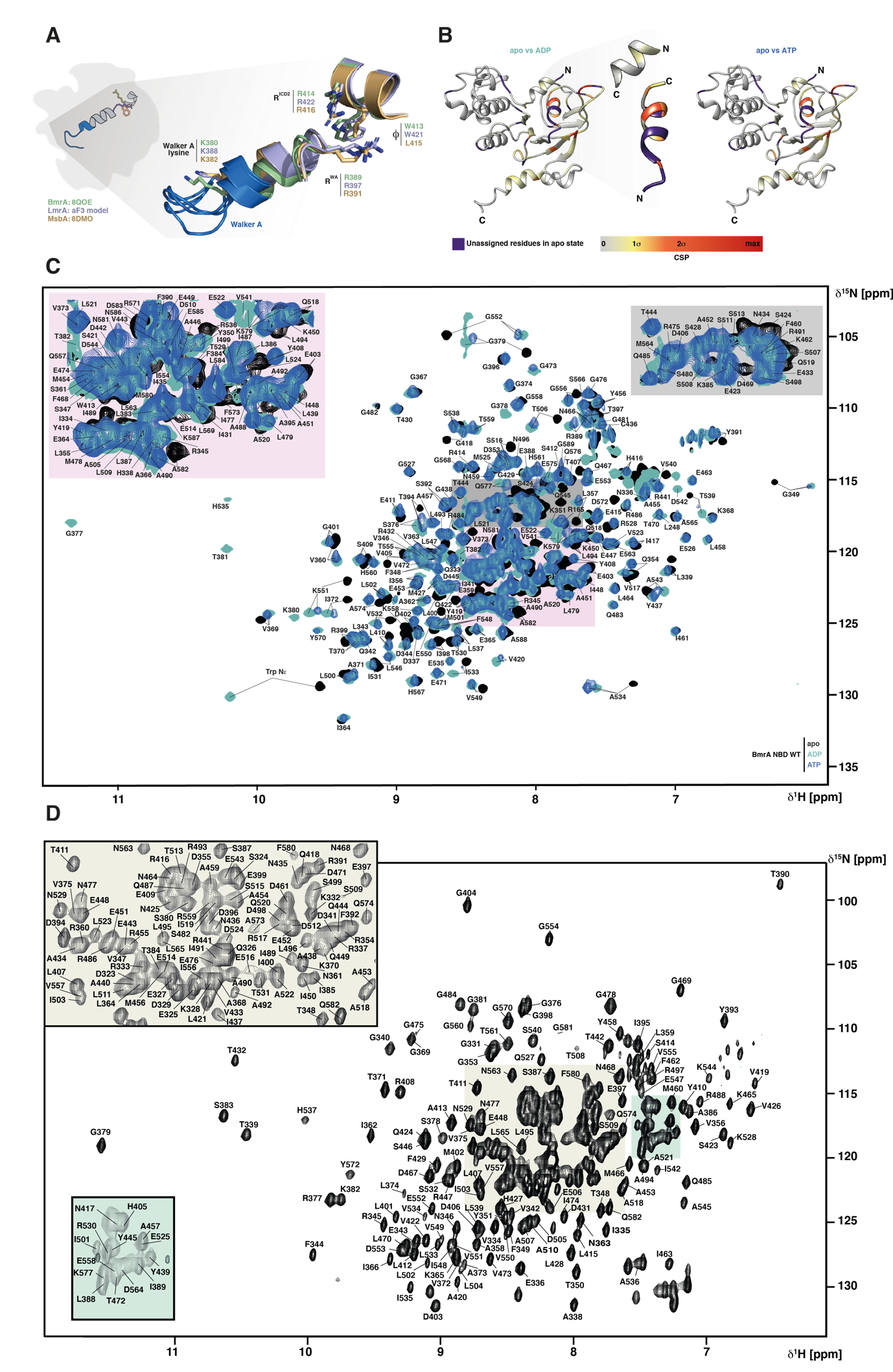


**Supplementary Figure 2:** **Structural conservation of hinge residues and nucleotide binding-induced chemical shift changes in the BmrA NBD.** (**A**) Structural overlay of the Walker A helix and hinge region in the NBD from *B. subtilis* BmrA (green, PDB ID: 8QOE^1^), *L. lactis* LmrA (blue, AlphaFold3 model), and *E. coli* MsbA (gold, PDB ID: 8DMO^3^) in the inward facing conformation. The conserved Walker A motif is shown in blue, the conserved Walker A lysine residue and the hinge residues are shown as coloured sticks. (**B**) Chemical shift perturbations (CSP) induced by the addition of 10 mM ADP (left) or 10 mM ATP (right) to ^15^N-labeled BmrA NBD WT mapped onto the cryoEM structure of BmrA (PDB: 6R81^2^). A zoom highlights the Walker A helix and the helix bearing the φ and R^ICD2^ residues, the color code reflects the CSP of residues comparing the apo vs ADP-bound state or vs the ATP-bound state. Residues without an amide backbone assignment in the apo state are colored in purple, these resonances appear upon ADP binding, suggesting stabilization of the nucleotide binding site, including the Walker A motif and the adjoining helix. CSP 1σ, 2σ and max values correspond to 0.01, 0.02 and 0.09 ppm respectively. (**C**) 2D–^1^H,^15^N–TROSY–HSQC spectrum of ^2^H, ^15^N–labeled BmrA–NBD in the apo state (black), with 10 mM ADP (teal) or 10 mM ATP (blue) were recorded at 298 K on a 600 MHz spectrometer equipped with a cryogenic triple resonance probe (Bruker GmbH, Karlsruhe, Germany), using samples with a concentration of 250 µM. To obtain the assignments of the apo and the ATP-bound states, we traced the chemical shift perturbations using our previously published backbone amide resonance assignment for BmrA bound to ADP^4^ (BMRB entry 51156). **(D)** 2D–^1^H,^15^N–TROSY–HSQC spectrum of ^2^H, ^15^N, ^13^C–labeled MsbA NBD bound to ADP showing its backbone assignment. Here, 91.7% of all non–proline residues successfully assigned. Sample was recorded on a 600 MHz spectrometer at 298 K using a 450 µM sample. The assignments are given in single letter code, following the numbering scheme for the *E. coli* full–length MsbA ABC transporter (uniprot ID: P60752, BMRB code: 52626).


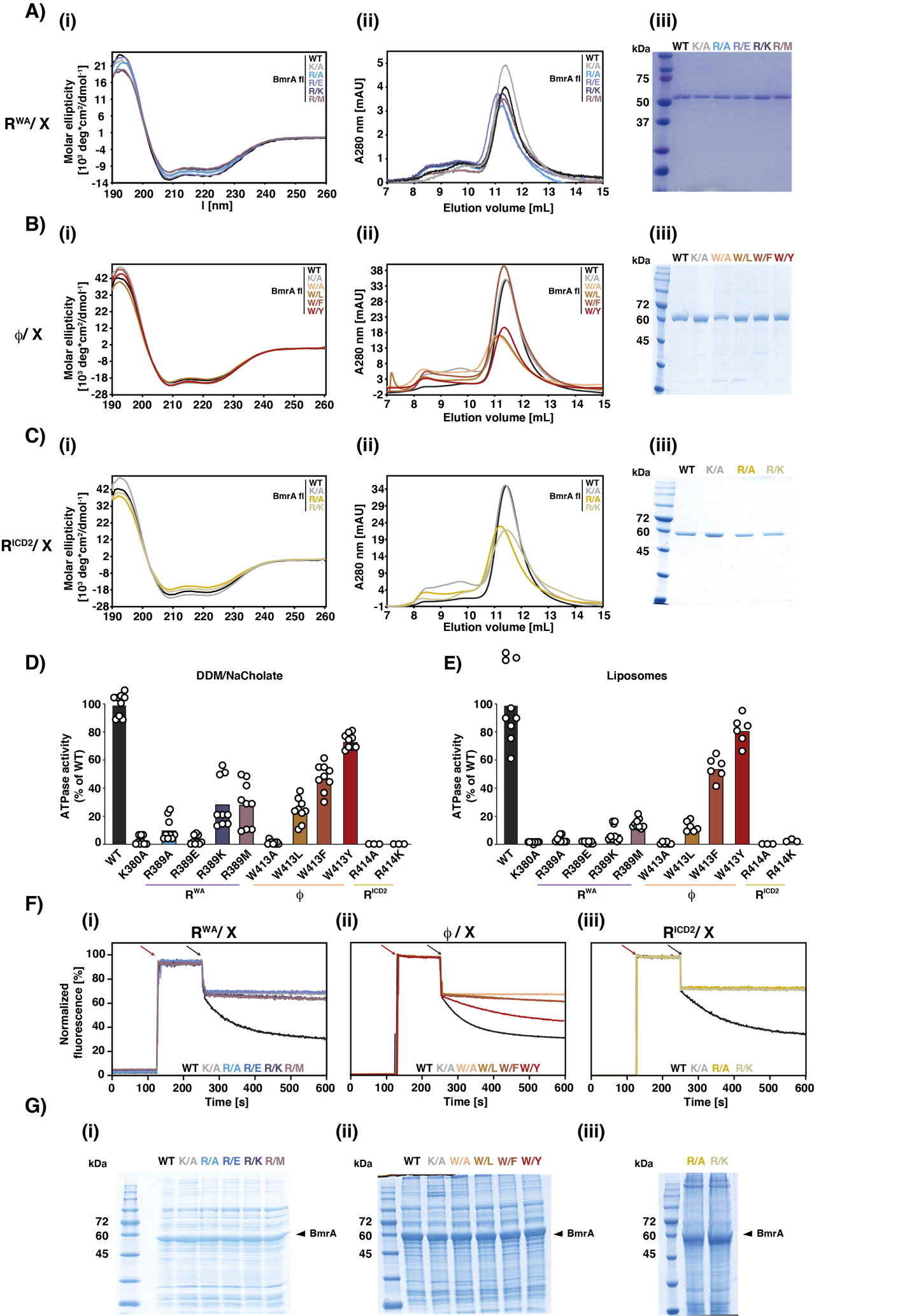


**Supplementary Figure 3: Role of hinge mutants for BmrA structural integrity and function.** (**A, B, C**) Circular dichroism spectra (i), size exclusion chromatography (ii) and SDS-PAGE of purified BmrA WT, Walker-A K380A, BmrA R^WA^ (R398X), φ (W413X) and R^ICD2^ (R414X) variants in DDM/cholate micelles. In (iii), 2.5 µg (A, B) or 1.0 µg (C) purified protein per lane were loaded (**D, E**) ATPase activity of BmrA variants in detergent micelles (DDM/NaCholate) (D) and reconstituted in liposomes prepared from *E. coli* polar lipid extract (E). All values were normalized to the WT activity. For WT, K380A and R389X constructs, results shown are the mean of three biological triplicates with three technical replicates each. For W413X constructs, results shown are the mean of two biological replicates with three technical replicates each. For R414X constructs, results shown are the mean of one biological replicates with three technical replicates. (**F**) Fluorescence-based doxorubicin transport assay with inside-out vesicles (IOVs) prepared from *E. coli* cells overexpressing *B. subtilis* BmrA (i) R^WA^ (R398X), (ii) φ (W413X) and (iii) R^ICD2^ (R414X) residue variants (see Figure 1E in main manuscript for a quantitative analysis). Red arrows at 125 s and black arrows at 250 s indicate addition of the fluorescent substrate doxorubicin and ATP, respectively. (**G**) All variants were expressed similarly as seen by SDS-PAGE of IOVs. The expected running height of BmrA is indicated by a black arrow.

**
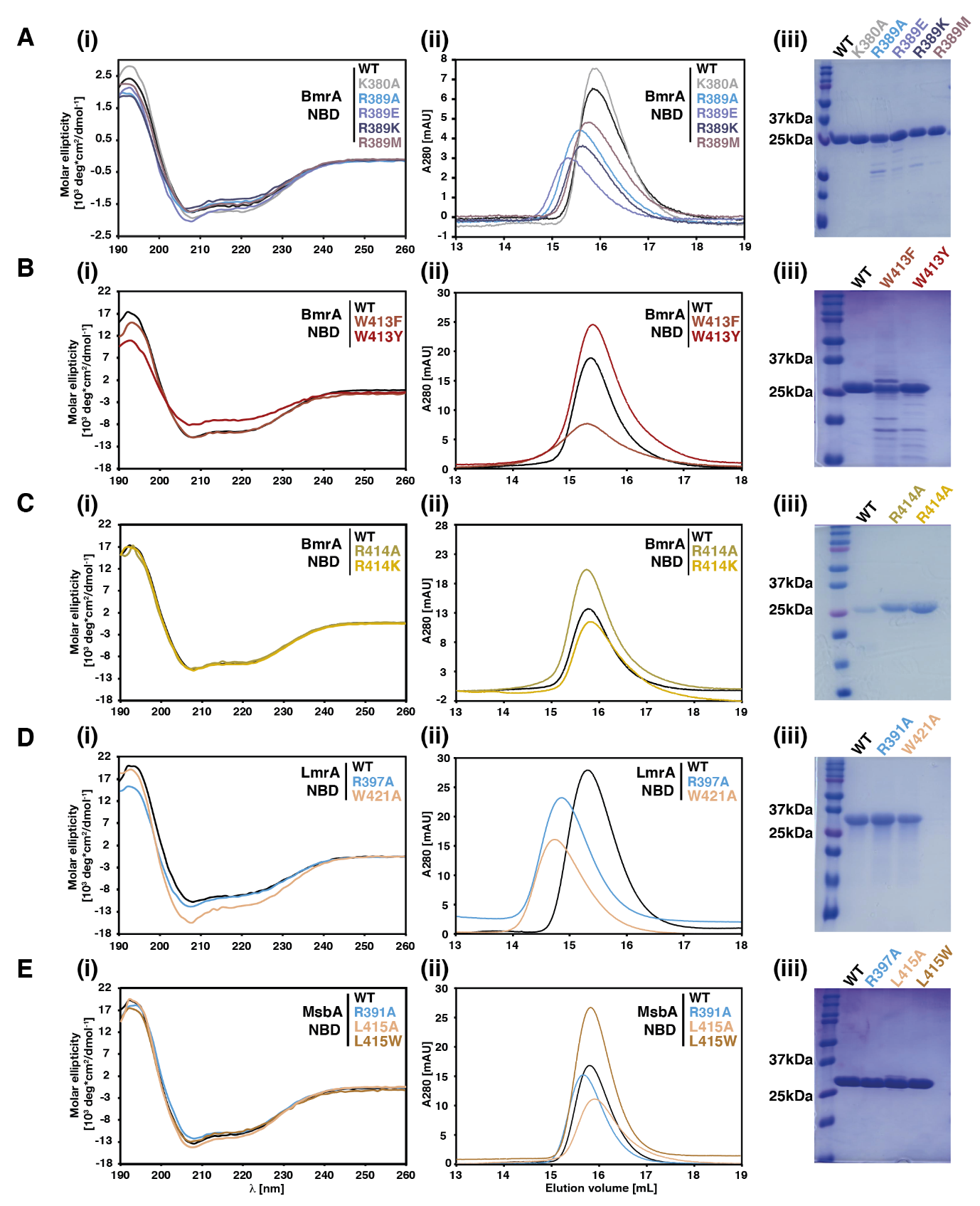
**

**Supplementary Figure 4: Structural integrity of the isolated NBDs from type IV ABC transporters containing hinge mutations. (A, B, C)** Circular dichroism spectra (i), Size exclusion chromatography profiles (ii) and SDS-PAGE (iii) of BmrA NBD with a mutation in residue R^WA^ (A), φ (B) or R^ICD2^(C), respectively. For reference, the BmrA NBD WT is included in (i-iii), and the Walker A K380A mutant in (i). (**D**) Circular dichroism spectra (i), Size exclusion chromatography profiles (ii) and SDS-PAGE (iii) of LmrA NBD WT and hinge mutants. (**E**) Circular dichroism spectra (i), size exclusion chromatography profiles (ii) and SDS-PAGE (iii) of MsbA NBD WT and hinge mutants.


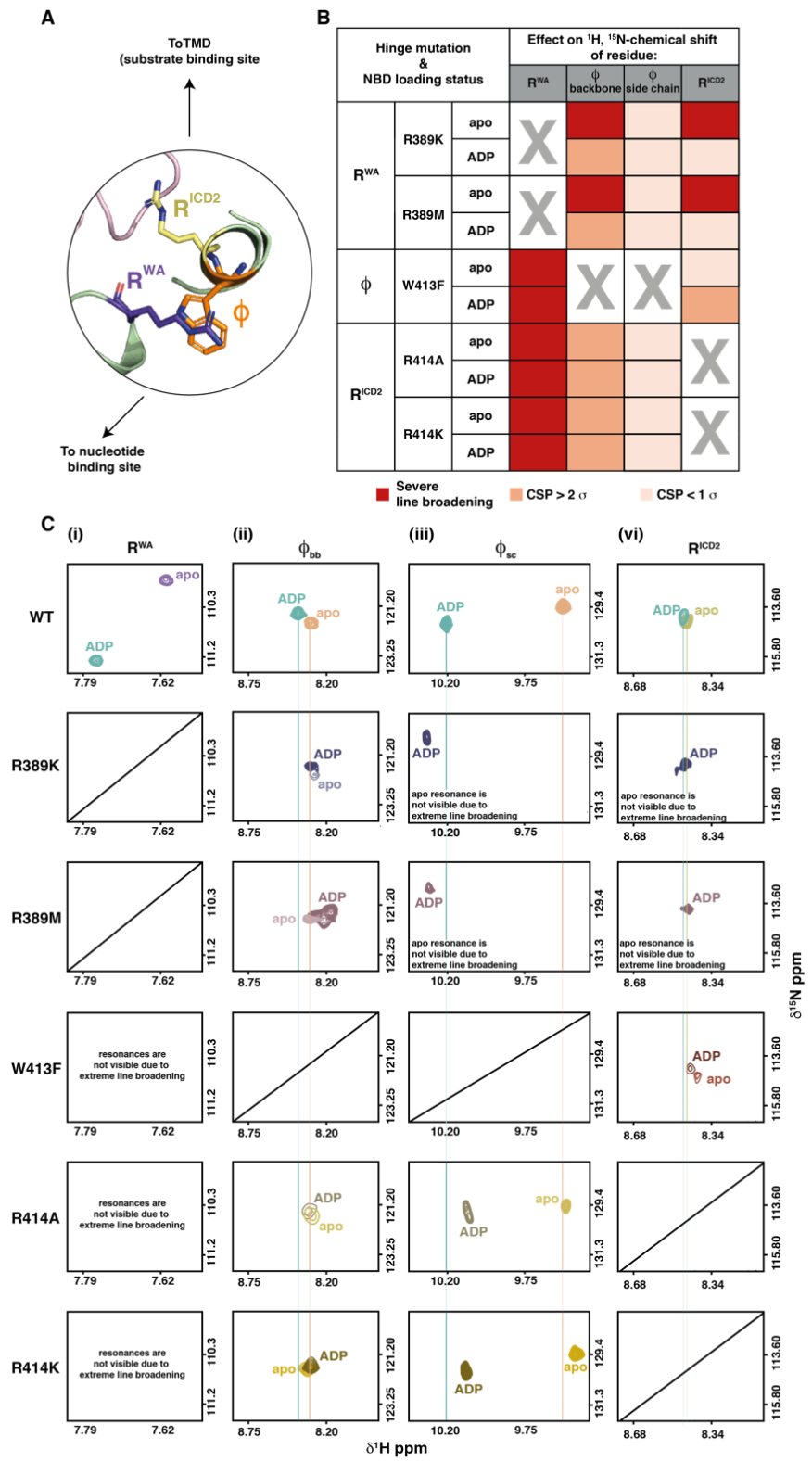


**Supplementary Figure 5:** **The hinge residues are structurally and dynamically coupled.** (**A**) Cartoon of communication hinge highlighting relative position of the three conserved residues in BmrA. (**B**) Table summarizing the data shown in (C), i.e., the nucleotide dependent effects on NMR chemical shift and line width for the ^1^H, ^15^N resonances of the hinge residues in the ^15^N-labeled BmrA NBD, upon mutating one of the three hinge residues. The respective mutated residue is crossed out. (**C**) Zoom into the ^1^H, ^15^N-HSQC to show the NH resonances for the three hinge residues in the WT NBD (top row) and upon mutating each of the hinge residues individually (bottom rows). Chemical shifts of WT NMR in the apo state and in the presence of 10 mM ADP are indicated shown as colored lines. Note that for residue φ, a tryptophan, both the backbone and sidechain indole NH resonances can be observed (denoted φ_bb_ and φ_sc_, respectively). The consequences of mutating either of the hinge residues on the chemical shift and linewidth compared to the WT is summarized in the table shown in (B).


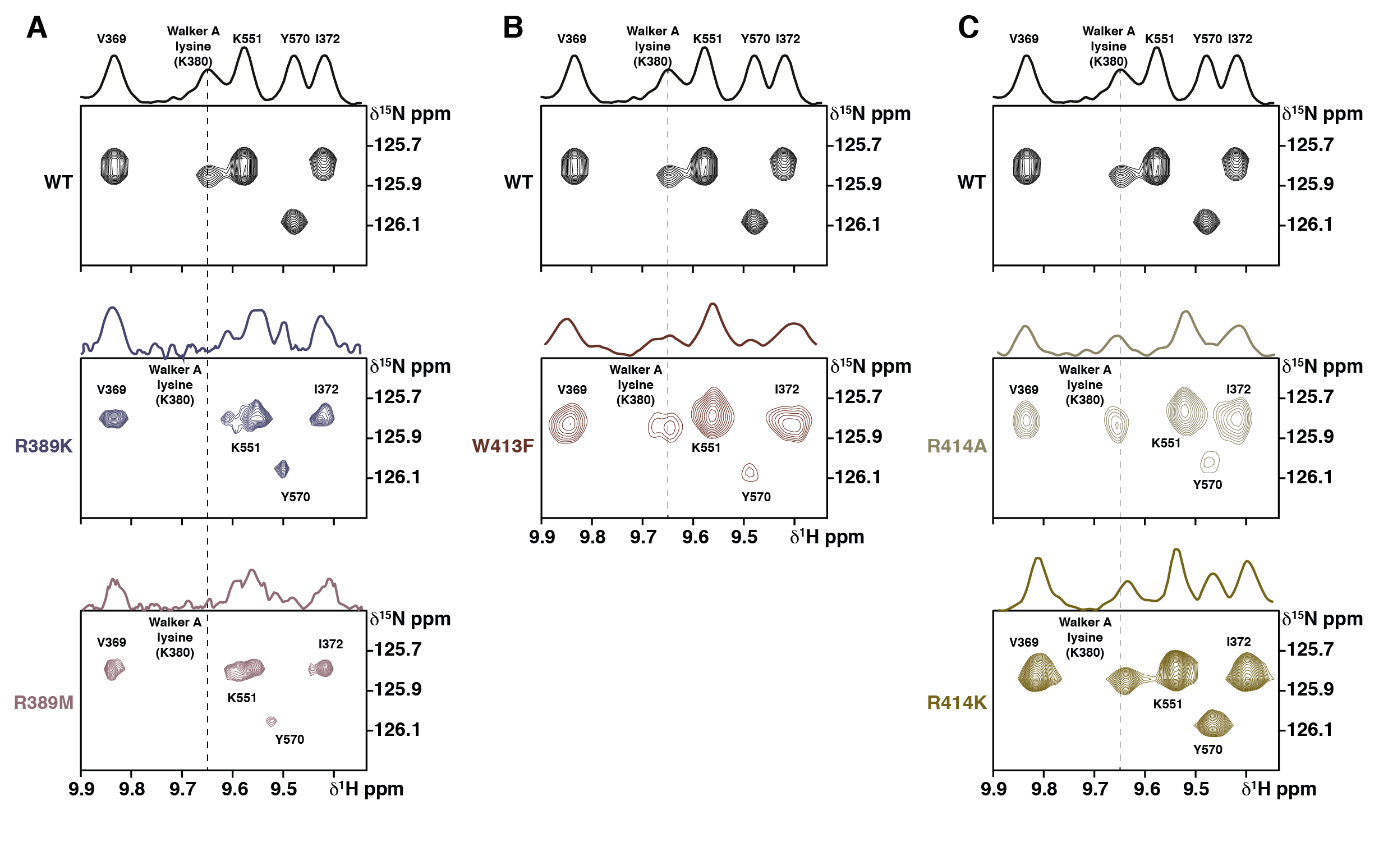


**Supplementary Figure 6: Effects of hinge mutations on the conserved Walker A lysine in the nucleotide binding site.** (**A, B, C**) Regions from the ^1^H, ^15^N HSQC NMR spectra of BmrA NBD WT and hinge mutants, focusing on the resonance of the conserved Walker A lysine K380, which becomes visible only in the presence of ADP. Thus, all spectra were recorded with 10 mM ADP at 298K on a 600 MHz spectrometer equipped with a cryogenic triple resonance probe (Bruker GmbH, Karlsruhe, Germany, using a 200 µM sample. For better visibility, the 1D projections are shown on top of each spectrum, and the ^1^H chemical shift of the K380 resonance in the WT NBD is marked with a dashed line. Upon mutation of residue R^WA^ (A), φ (B) or R^ICD2^ (C), chemical shift changes and line broadening for K380 occurred, showing that nucleotide binding site and hinge are structurally and dynamically coupled.

**
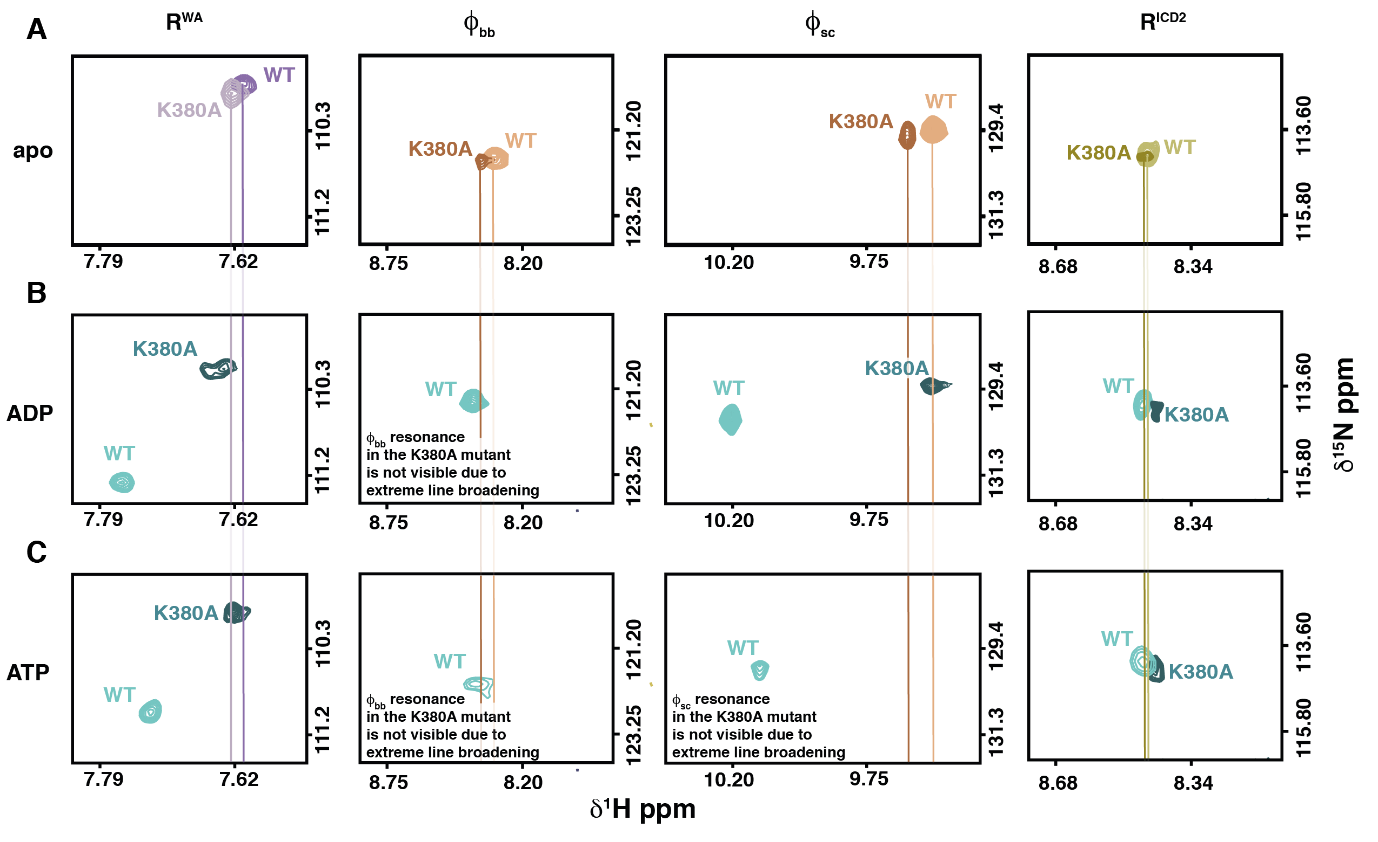
**

**Supplementary Figure 7: Effect of Walker A mutant K380A on hinge residues.** (**A**) Comparison of chemical shifts of hinge residues between BmrA NBD WT and Walker A K380A mutant in the absence of nucleotides showing that mutation of the nucleotide binding site alone is sensed by the hinge residues. (**B, C**) Comparison of chemical shifts of hinge residues between BmrA NBD WT and Walker A K380A mutant in the presence of 10 mM ADP (B) and ATP (C). In some cases, mutation of the Walker A residue led to severe line broadening in the hinge. All spectra were recorded on a 600 MHz spectrometer equipped with a cryogenic triple resonance probe (Bruker GmbH, Karlsruhe, Germany) at 298K using a 200 µM sample.

**
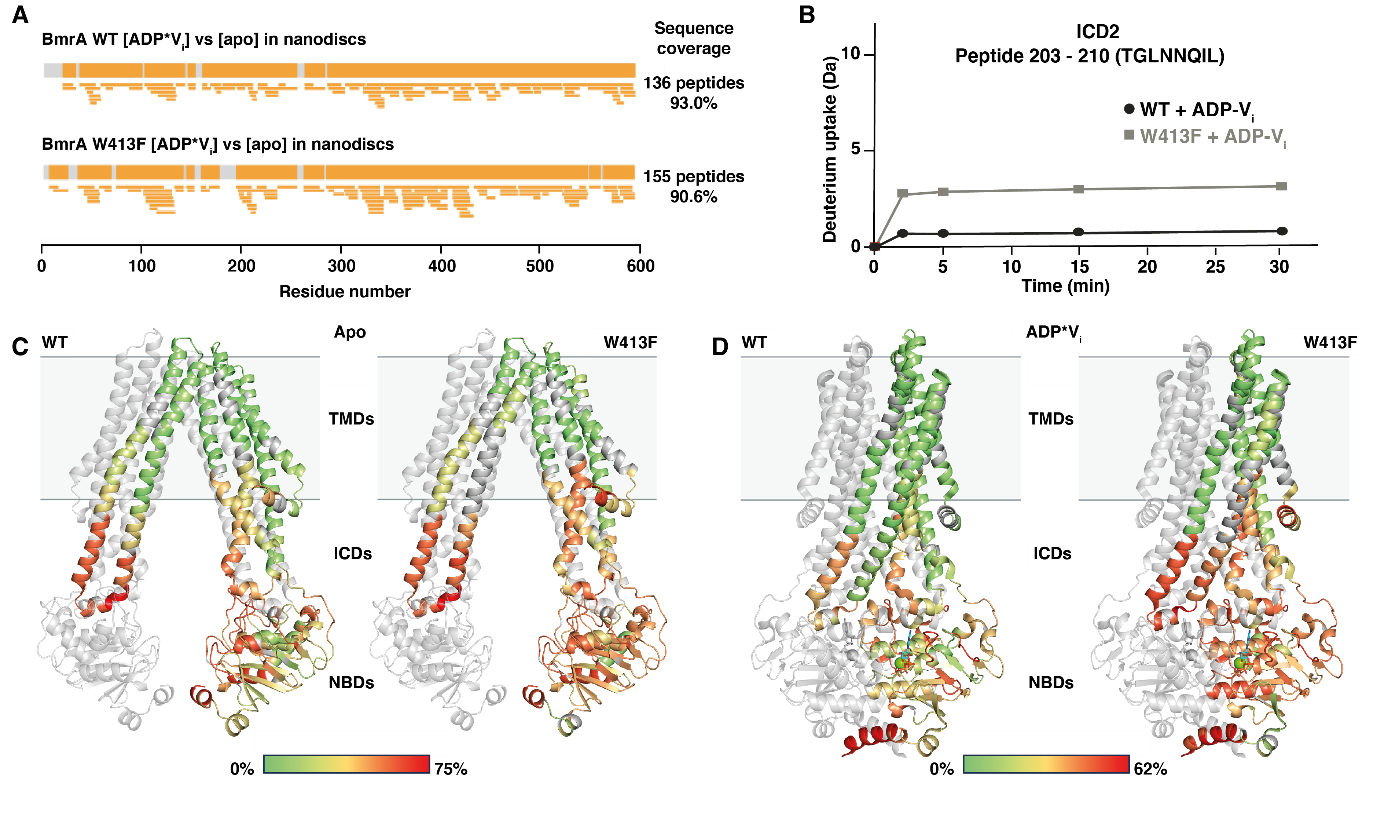
**

**Supplementary Figure 8. Impact of BmrA hinge W413F mutation on transporter dynamics in lipid nanodiscs followed by HDX-MS.**(**A**) Sequence coverage map of BmrA WT (upper panel) and W413F (lower panel) showing peptides (orange bars) shared between the apo- and the ADP*V_i_ trapped states (upon incubation with 10 mM Mg^2+^, 10 mM ATP and 1 mM Vi).  (**B**) The deuterium uptake for a peptide (residues 203–210 (TGLNNQIL)) within ICD2 after incubation with ATP-Vi exhibits significantly reduced HDX in the ADP*V_i_ trapped state for the WT (black), relative to the hinge mutant (grey) (*p-value* > 0.05 using Peptide-level significance statistical tests^5^). Deuteration uptakes are presented as mean values of two or three technical replicates +/- SEM, for WT or W413F respectively. (**C**) HDX after 30 min deuteration for BmrA WT (left) and hinge mutant (right) in the apo state. HDX was plotted on the cryoEM structure of BmrA in the open inward state (PDB: 8QOE^1^). For clarity, one BmrA monomer was kept transparent, while the second monomer was colored in a deuteration scale ranging from 0 to 75%, with 75% representing the maximum deuteration observed under these conditions. Uncovered peptides are represented in dark gray. (**D**) HDX after 30 min deuteration for BmrA WT (left) and hinge mutant (right) in the ADP*Vi trapped state plotted on the cryoEM structure of BmrA in the OF state (PDB: 7OW8^2^). One BmrA monomer is transparent, while the second monomer is colored in a deuteration scale ranging from 0 to 62%, with 62% representing the maximum deuteration observed under these conditions.


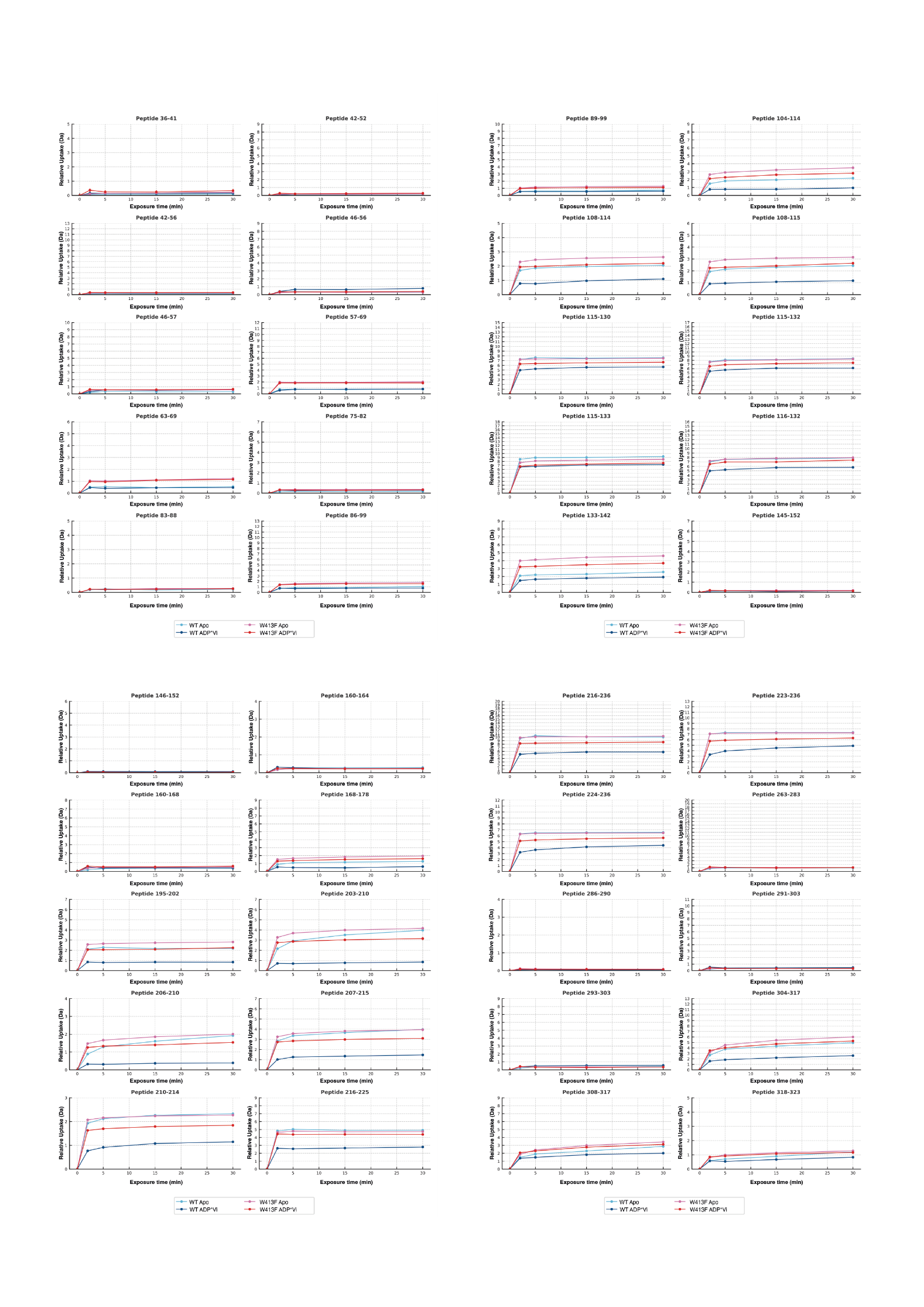


**Supplementary Figure 9:** **Kinetics of HDX for the common peptides identified in both the WT and the W413F mutant in nanodiscs.**  Peptides are shown for the apo and ADP*V_i_ trapped conditions. These data were used to generate supplementary table 4. Light blue and dark blue traces, apo and ADP*V_i_ states of the WT protein, respectively; Pink and red traces, apo and ADP*V_i_ states of the W413F mutant, respectively.


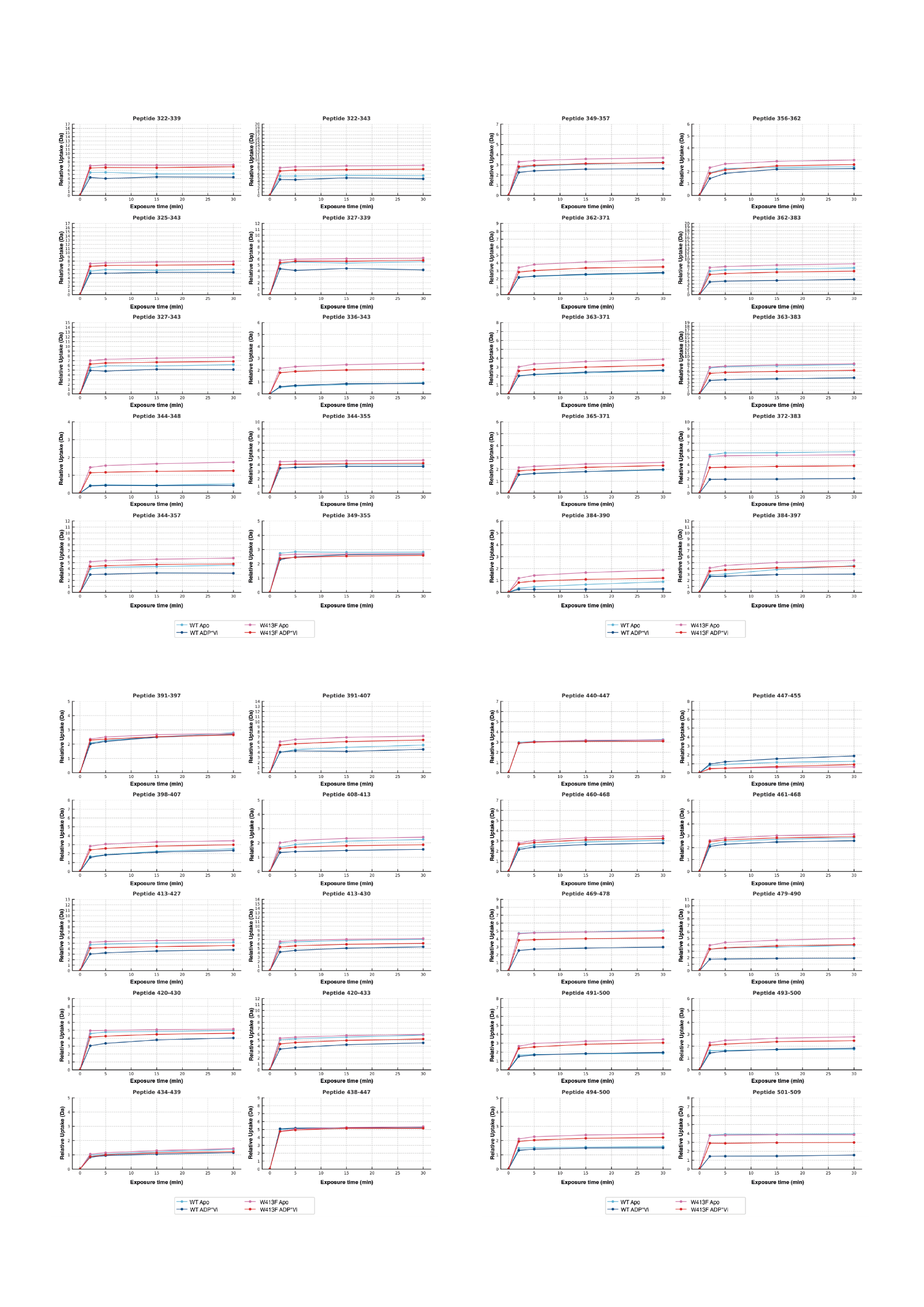


**Supplementary Figure 9 (continued)**


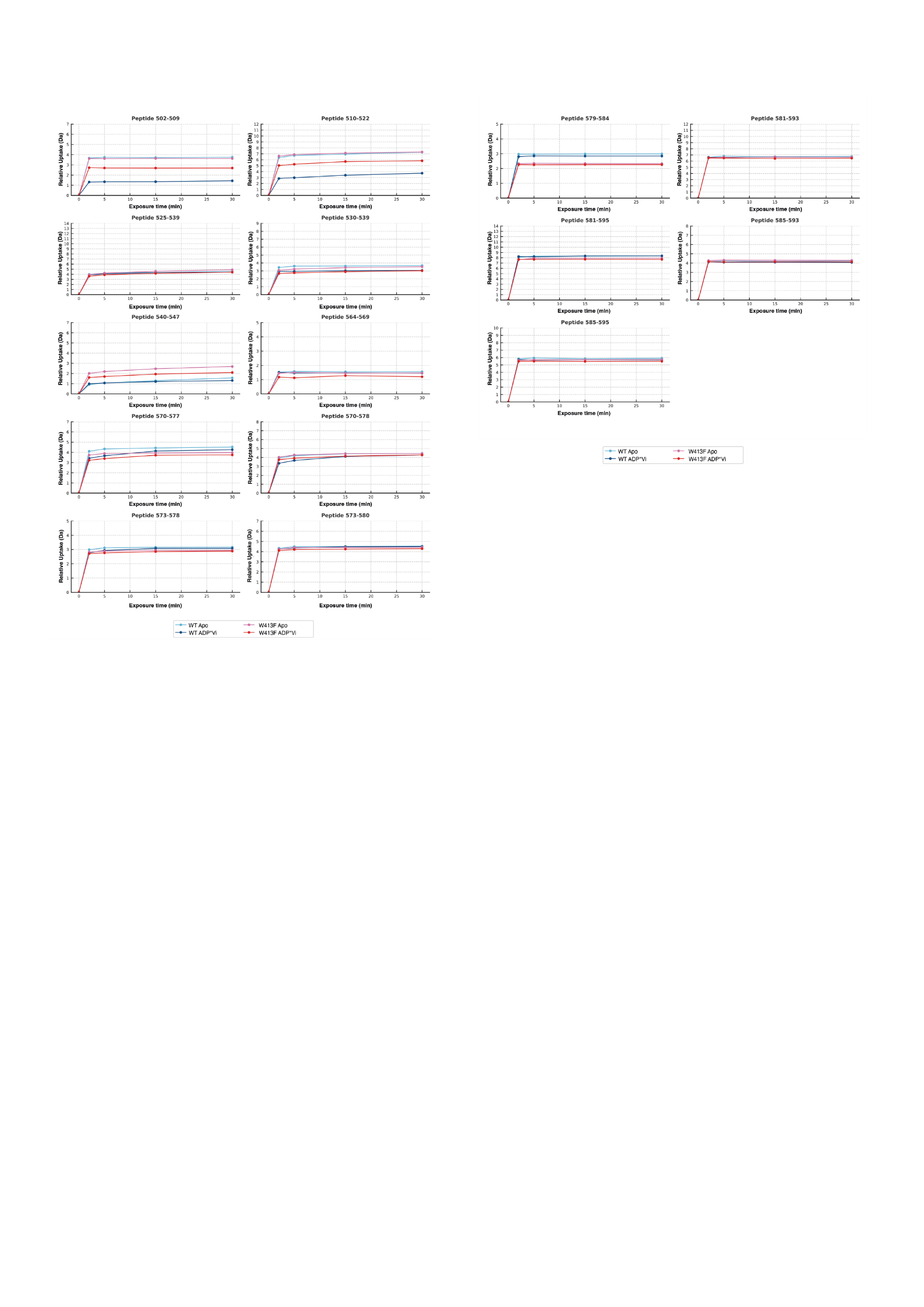


**Supplementary Figure 9 (continued)**

**
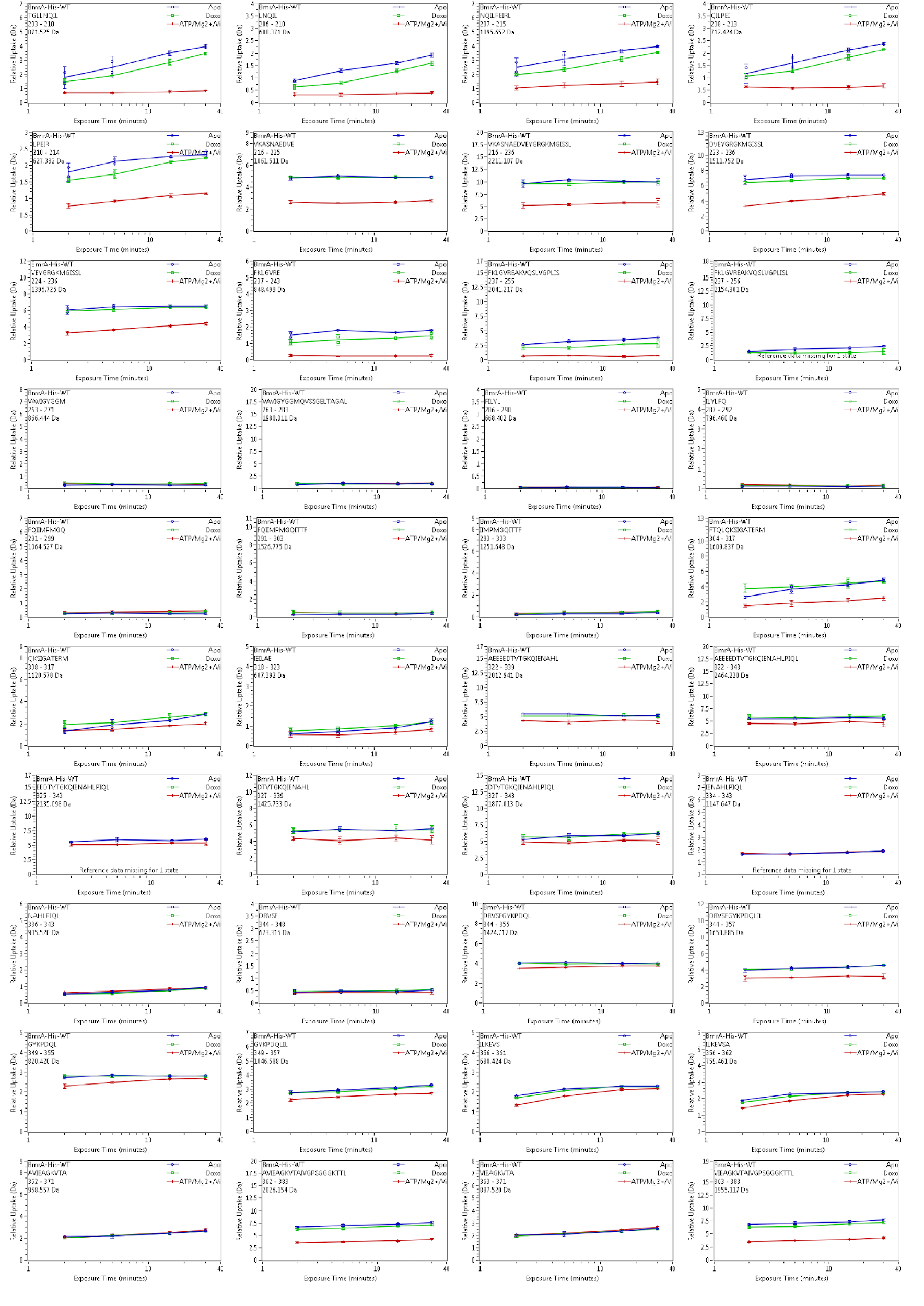
**

**Supplementary Figure 10:** **Kinetics of deuteration of different peptides from the WT transporter in nanodiscs.** For the majority of peptides from WT in three conditions (apo, ADP*V_i_ trapped and doxorubicin bound), a classic EX2 regime was observed. The W413F mutant mostly replicated the behavior seen for the WT protein (see Supplementary Figure 11). However, some of the peptides showed an EX1 regime, notably in ICD1 (between residues 108 to 132), ICD2 (between residues 189 to 255), the linker region between the TMD and the NBD (between residues 304-343) and the alpha-helical domain (between residues 480-500). Please see supplementary figure 12 for an example of EX1 regime for the deuteration kinetics of a peptide from ICD2.

**
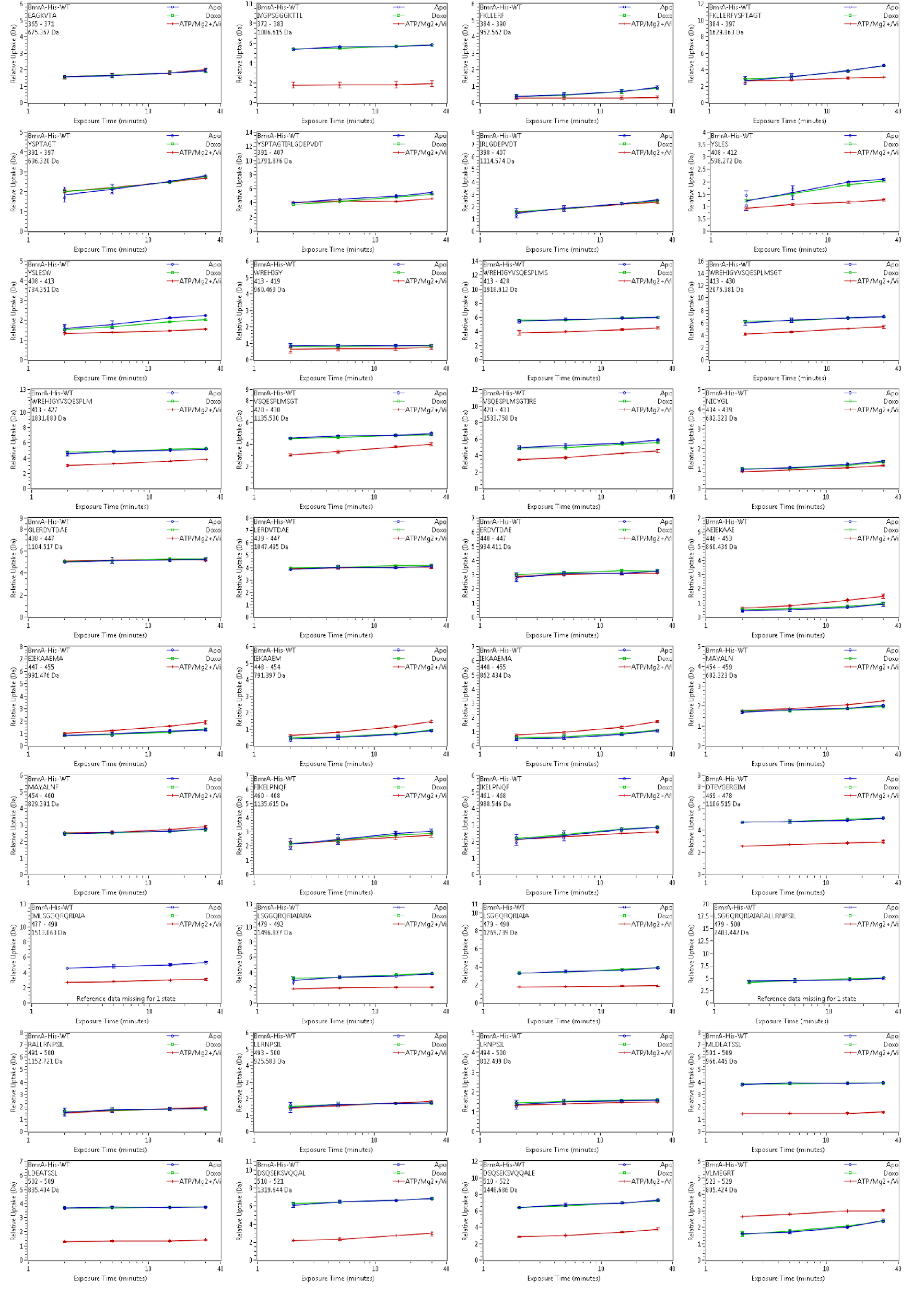
**

**Supplementary Figure 10 (continued)**

**
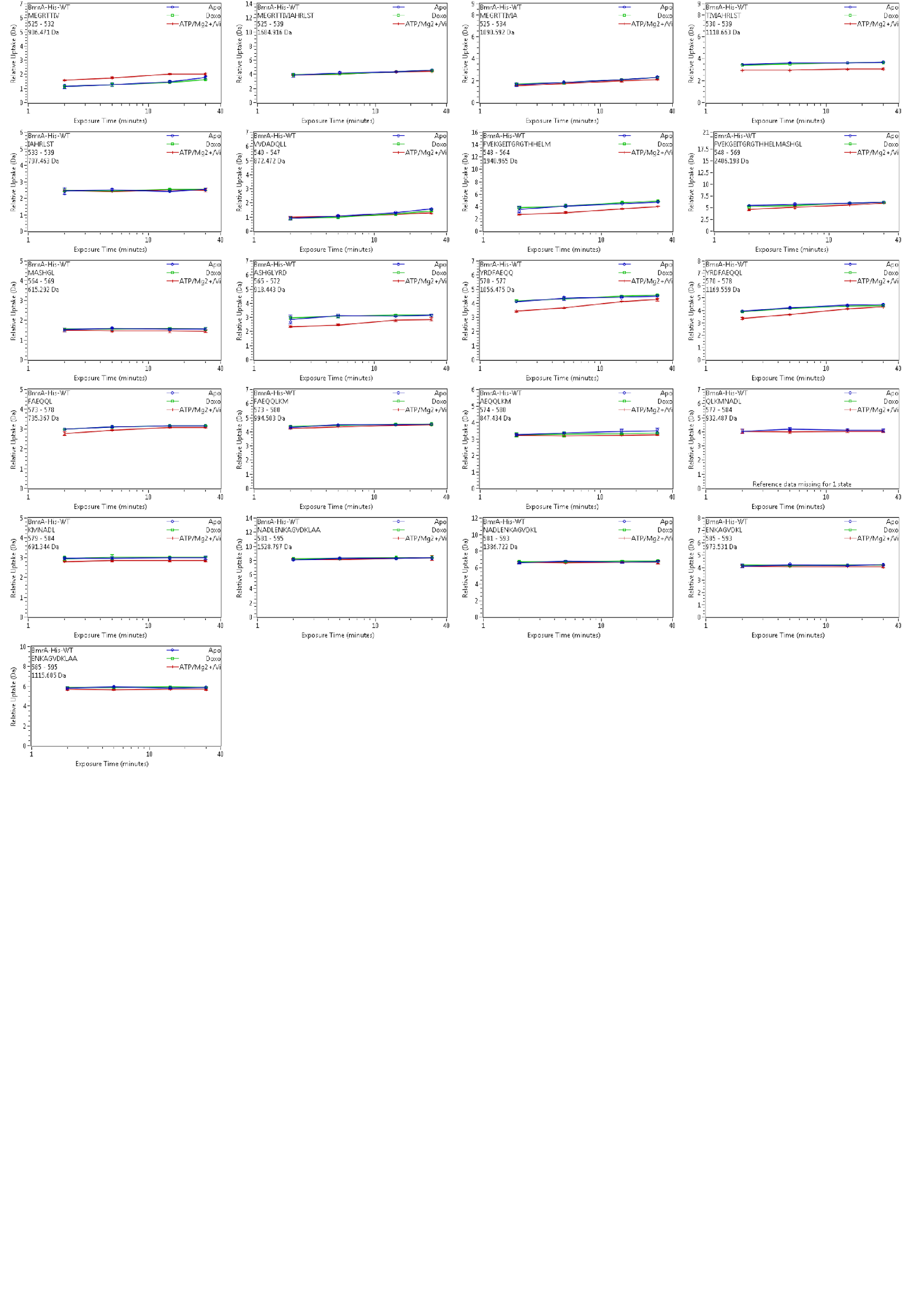
Supplementary Figure 10 (continued)**

**
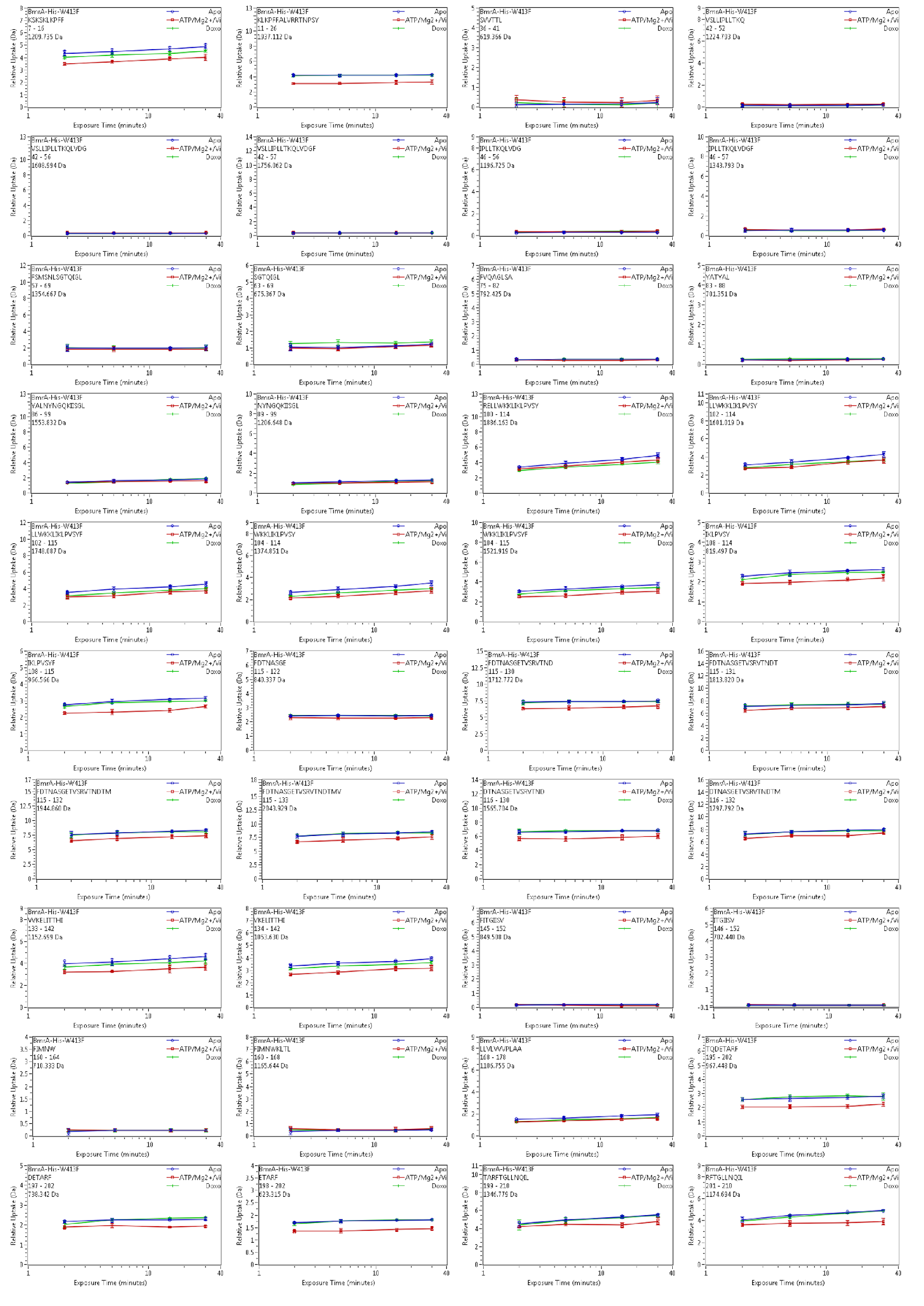
**

**Supplementary Figure 11: Kinetics of deuteration of different peptides from the W413F hinge mutant in nanodiscs.** For the majority of peptides from the mutant in three conditions (apo, ADP*V_i_ trapped and doxorubicin bound), a classic EX2 regime was observed, mostly replicating the behavior seen for the WT protein (see Supplementary Figure 10).

**
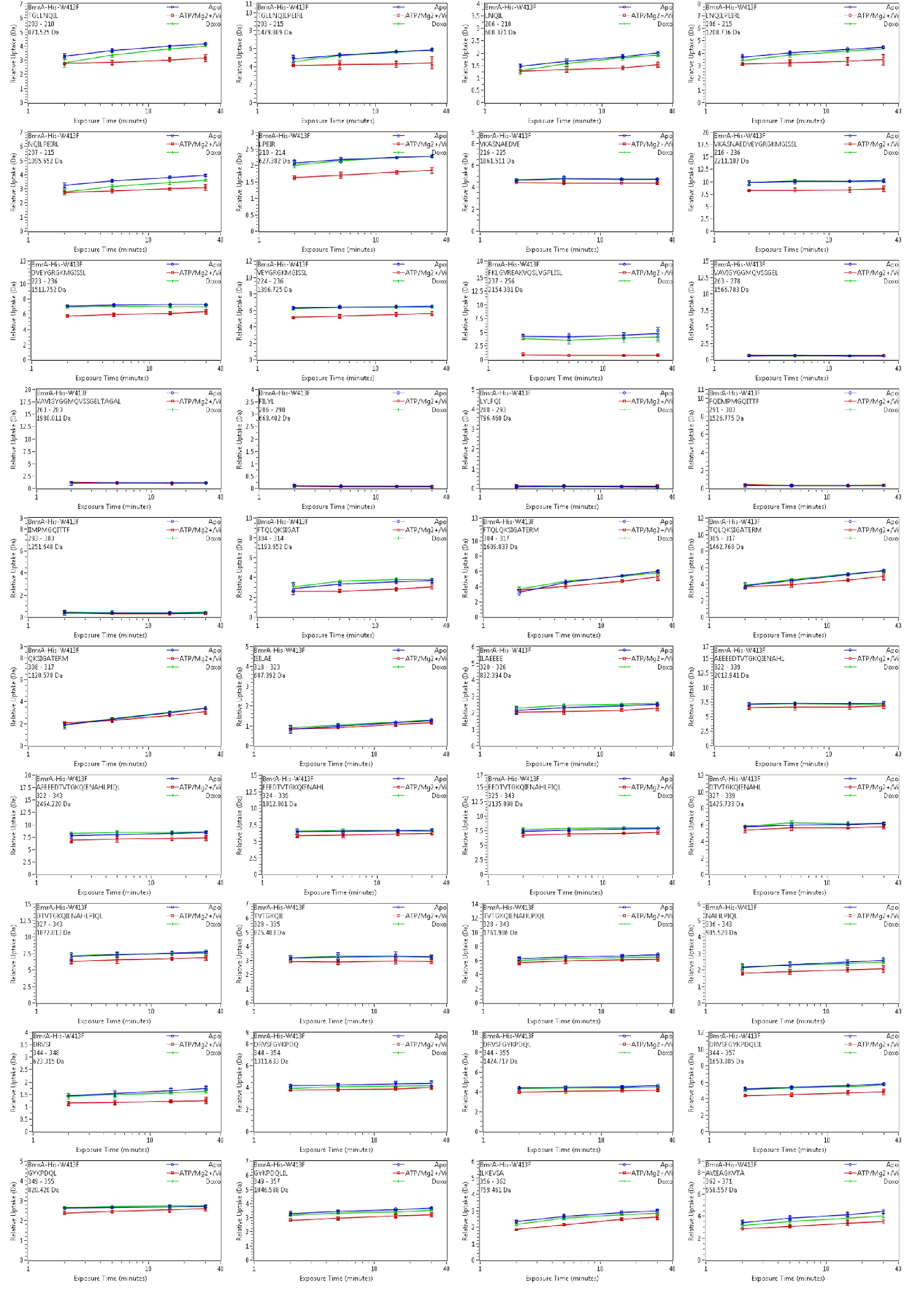
**

**Supplementary Figure 11 (continued)**

**
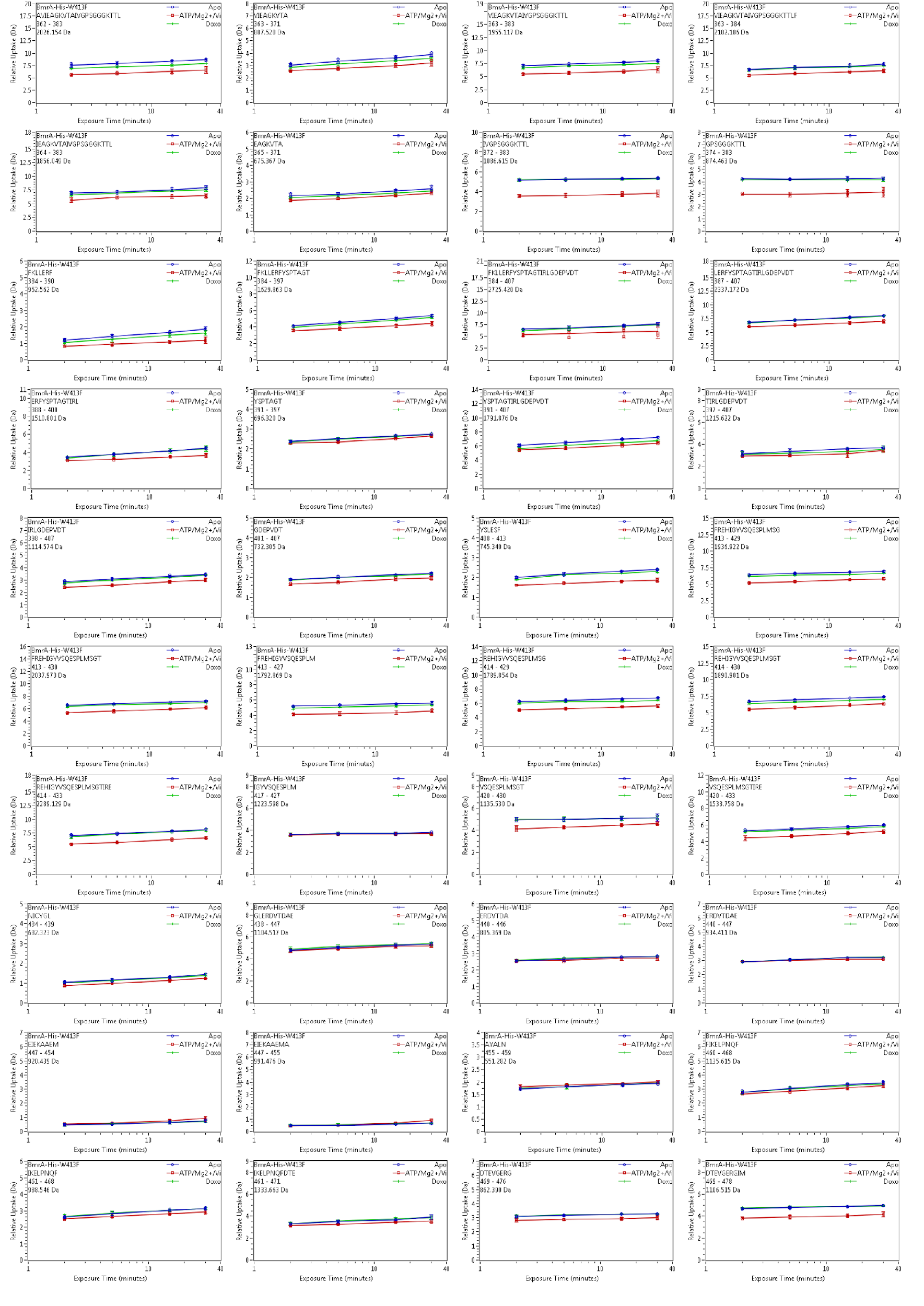
**

**Supplementary Figure 11 (continued)**

**

**

**Supplementary Figure 11 (continued)**

WT (ADP*Vi)

WT (doxo)

WT (apo)


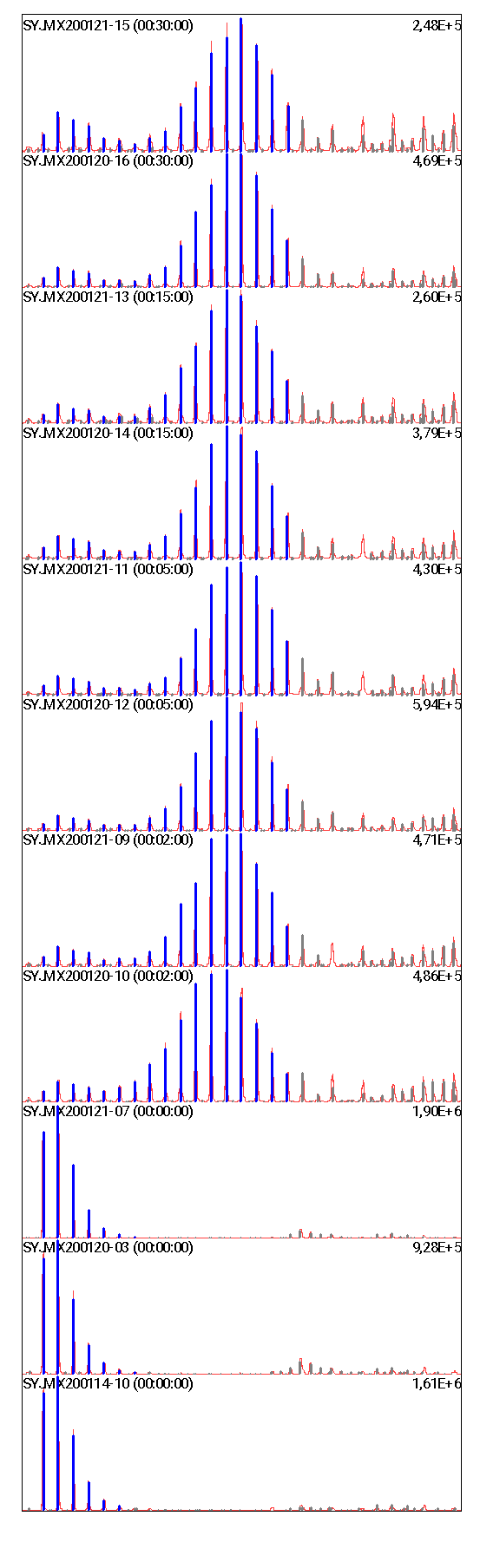

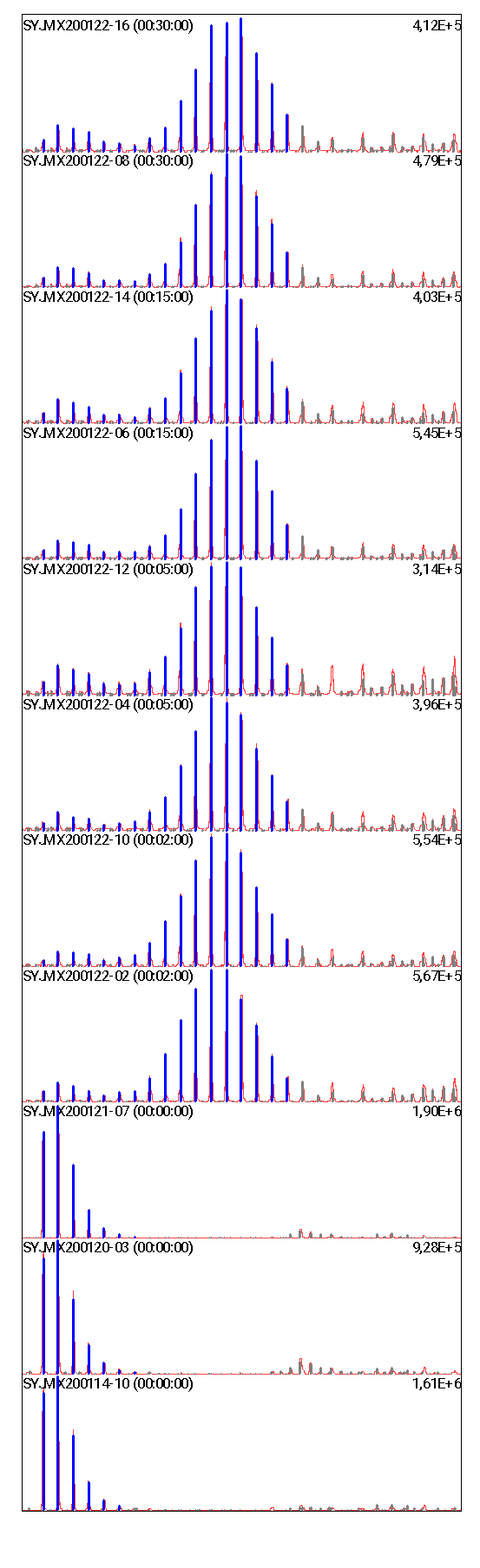

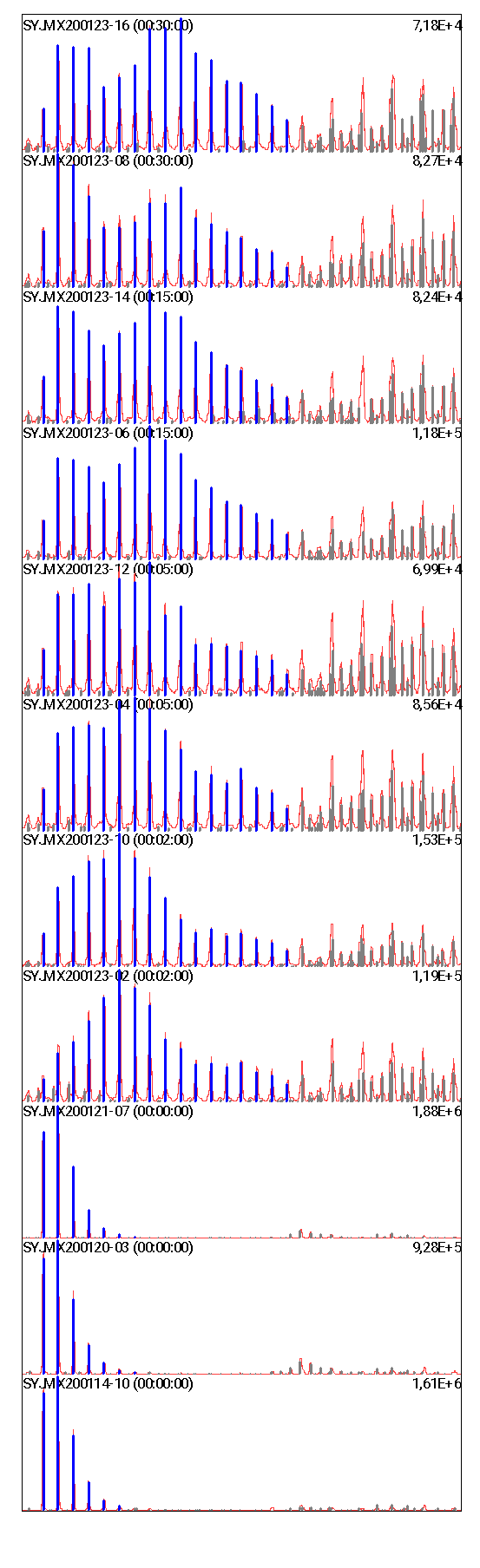


**Supplementary Figure 12:** **Example of isotopic distribution of the peptide 216-236 (ICD2) from the WT or the W413F mutant transporter in nanodiscs in the three tested conditions (apo, ADP*V_i_ trapped and doxorubicin bound)**. Peptides are showing an EX1 regime (i.e., bimodal isotopic envelope). Measurements were taken at 0 min (bottom, triplicate), 2min, 5min, 15min, 30min (from bottom to top, duplicates). Red and blue lines correspond to raw data and peaks picked by DynamX, respectively.

W413F (apo)

W413F (doxo)

W413F (ADP*Vi)


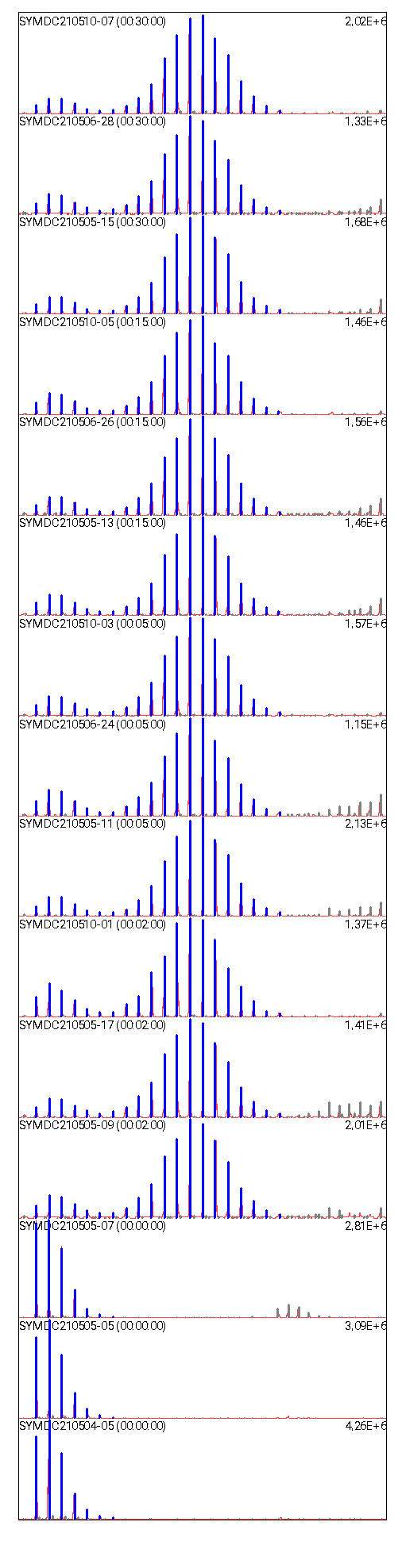

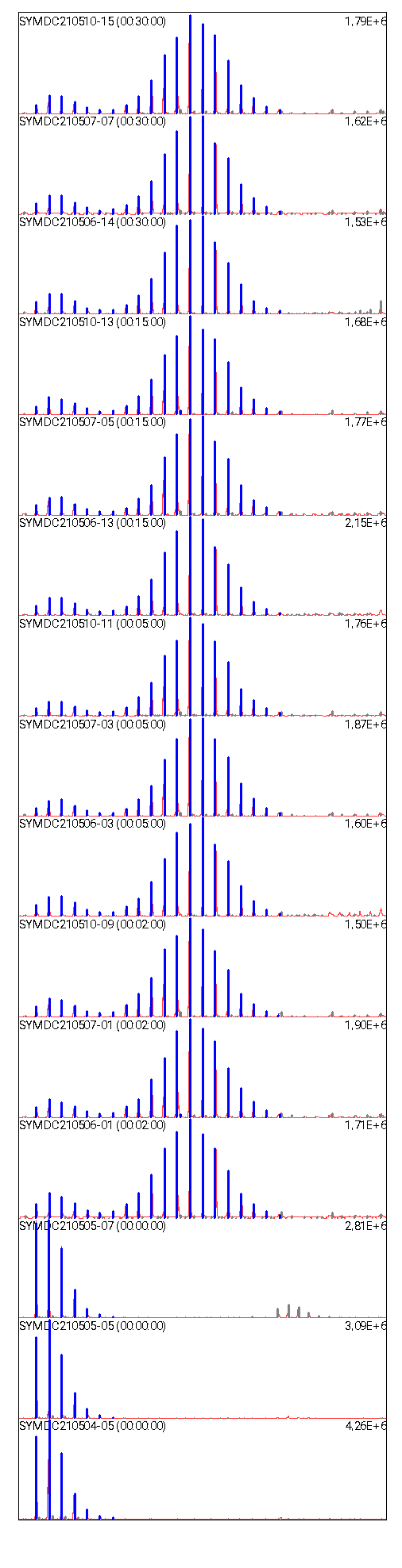

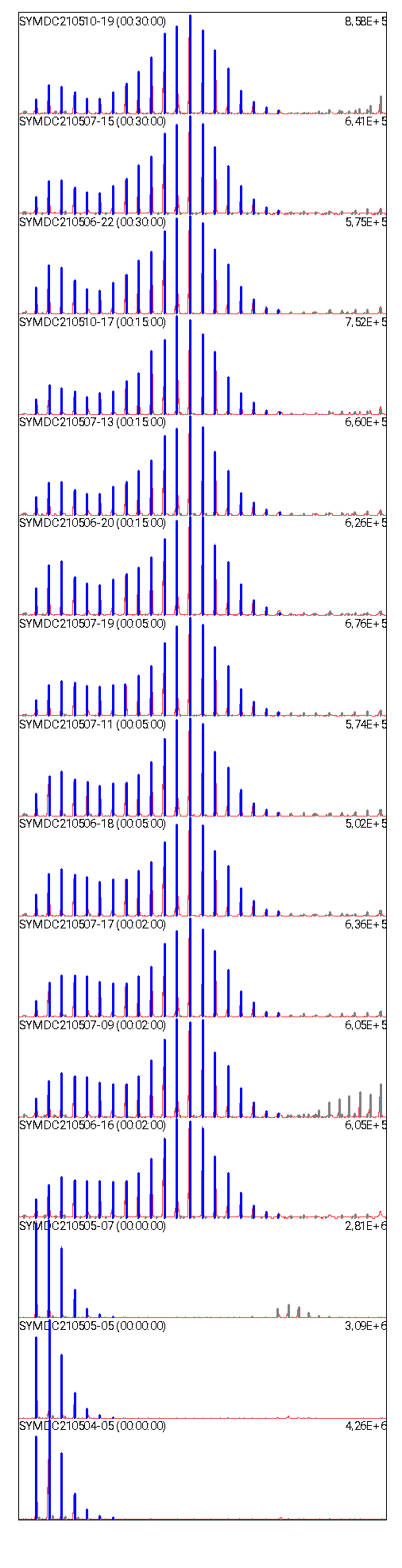


**Supplementary Figure 12 (continued)**

**
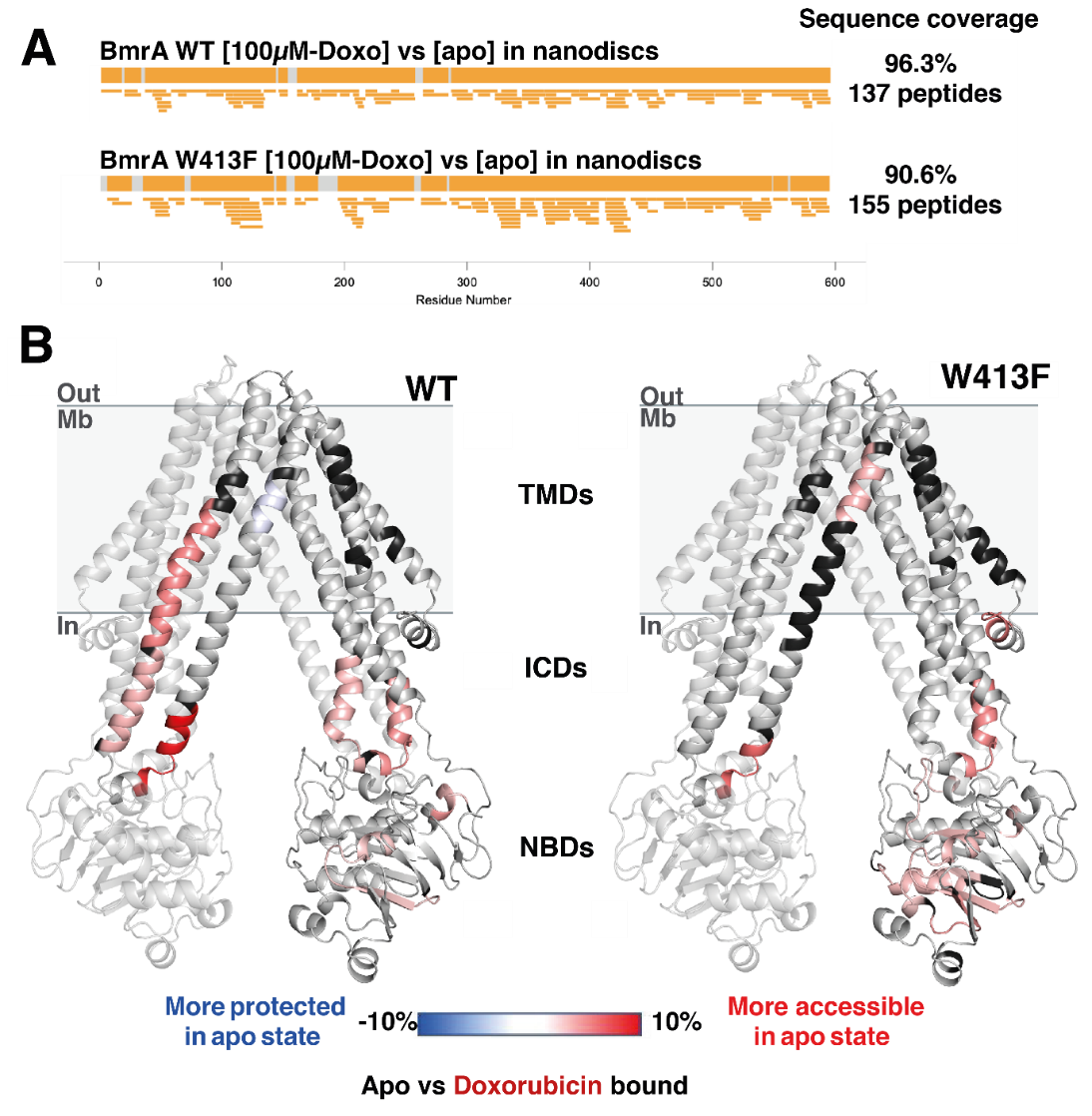
**

**Supplementary Figure 13: Hydrogen Deuterium Exchange coupled with mass spectrometry on BmrA in lipid nanodiscs in the presence of doxorubicin.** (**A**) Sequence coverage map of BmrA WT (upper panel) and W413F (lower panel) showing peptides (orange bars) shared between the apo- and the doxorubicin-bound states (upon addition of 100 µM doxorubicin and incubation for a minimum of 15 min at 20°C). Similar peptide coverage as for the apo and ADP*V_i_ trapped transporter was achieved. (**B**) HDX-MS in the absence and presence of doxorubicin. Shown are the differences between the apo and doxorubicin-bound states for BmrA WT (left) and hinge mutant W413F (right) after 15 min deuteration, plotted on the cryoEM structure of BmrA in the inward-facing state (PDB: 8QOE^1^). In the hinge mutant, noticeably reduced effects are observed. Data represent mean values of two (WT) or three (W413F) technical replicates. For clarity, one BmrA monomer was kept transparent, while the second monomer was colored in a deuteration scale ranging from -10 to 10 %, with 10 % representing the maximum deuteration observed under these conditions. Peptides with no significant deuteration differences (*p-value* < 0.05 using Peptide-level significance statistical tests^5^) are shown in light gray and non-covered peptides in black.


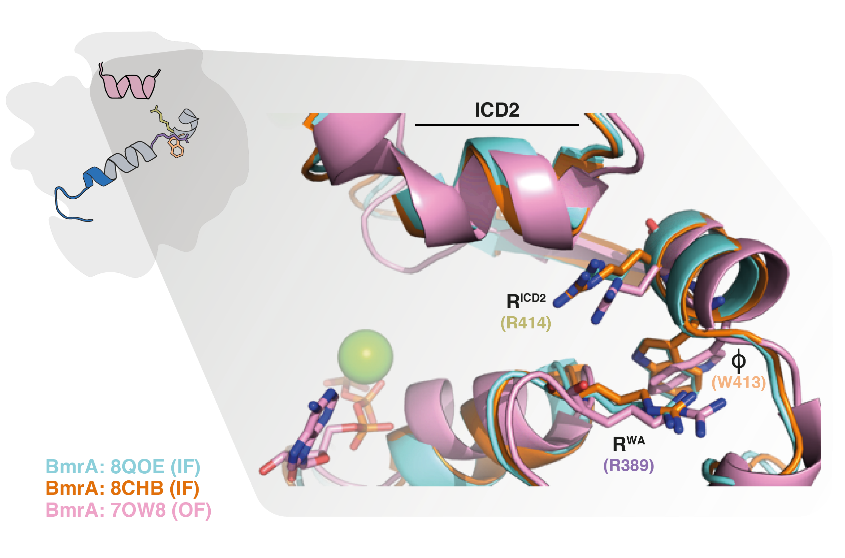


**Supplementary Figure 14: Comparison of the hinge region in the inward and outward facing conformations of BmrA.** Overlay of the hinge region in three available structures of BmrA. Two structures were solved in the inward facing (IF) conformation (PDBs: 8QOE^1^, 8CHB^1^) and the third structure was determined in the outward facing (OF) conformation bound to MgATP using the catalytically inactive mutant E504A (7OW8^2^). These three structures were selected after visual inspection of the electron densities in the six BmrA structures available in the PDB at this site, allowing the proper positioning of the side chains of the hinge residues in the modelled structures. In the three structures, the R^WA^, φ and R^ICD2^ side chains occupy highly similar positions in agreement with the observed electron densities, resulting in a highly similar side chain orientation of residue R^ICD2^ pointing towards the coupling helix of the protomer in *trans*.

**Supplementary Tables**

**Supplementary Table 1: Thermal stability of BmrA, LmrA and MsbA NBD communication hinge mutants.** Melting temperatures (*T_m_*) of ABC transporter NBDs were determined without nucleotides (apo) and in the presence of 10 mM ATP or ADP using a SYPRO Orange fluorescence assay. Results are shown as mean ± standard deviation. All measurements were performed with two biological replicates.

| **Protein construct** | | ***T_m,_* _apo_**  **(ºC)** | ***T_m,_* _MgADP_**  **(ºC)** | ***T_m,_* _ATP_**  **(ºC)** | ***T_m,_* _MgATP_**  **(ºC)** |
| --- | --- | --- | --- | --- | --- |
| **BmrA NBD** | **WT** | 41.1 ± 0.2 | 45.8 ± 0.5 | 44.0 ± 0.1 | 44.3 ± 0.1 |
|  | **K380A** | 41.0 ± 0.1 | 40.5 ± 0.1 | 40.3 ± 0.1 | 40.4 ± 0.2 |
|  | **R389A** | 33.8 ± 0.5 | 40.6 ± 0.4 | 39.6 ± 0.3 | 39.9 ± 0.3 |
|  | **R389E** | 33.6 ± 0.1 | 37.5 ± 0.2 | 37.1 ± 0.1 | 37.4 ± 0.3 |
|  | **R389K** | 34.9 ± 0.3 | 41.0 ± 0.3 | 39.3 ± 0.1 | 39.5 ± 0.2 |
|  | **R389M** | 36.6 ± 0.3 | 40.5± 0.1 | 39.8 ± 0.1 | 39.9 ± 0.4 |
|  | **W413A** | Non purifiable protein | | | |
|  | **W413L** | Non purifiable protein | | | |
|  | **W413F** | 45.9 ± 0.3 | 51.2 ± 0.4 | 51.0 ± 0.2 | 50.1 ± 0.3 |
|  | **W413Y** | 36.8 ± 0.4 | 46.7 ± 1.0 | 47.0 ± 0.3 | 44.2± 0.4 |
|  | **R414A** | 39.0 ± 0.1 | 46.8 ± 0.1 | 44.5 ± 0.3 | 43.7 ± 0.2 |
|  | **R414K** | 39.5 ± 0.1 | 46.2 ± 0.2 | 45.1 ± 0.4 | 43.7 ± 0.1 |
| **LmrA NBD** | **WT** | 41.2 ± 1.7 | 46.1 ± 0.1 | 44.1 ± 0.9 | 46.8 ± 0.1 |
|  | **R397A** | 34.9 ± 0.8 | 33.9 ± 1.5 | 33.5 ± 1.3 | 35.6 ± 2.3 |
|  | **W421A** | 32.3 ± 0.1 | 33.1 ± 1.6 | 31.8 ± 0.6 | 33.6 ± 0.1 |
| **MsbA NBD** | **WT** | 47.2 ± 0.1 | 51.0 ± 0.1 | 52.1 ± 1.1 | 50.0 ± 0.3 |
|  | **R391A** | 32.0 ± 0.5 | 44.8 ± 0.4 | 45.1 ± 0.1 | 43.8 ± 2.5 |
|  | **L415A** | 37.6 ± 0.9 | 45.7 ± 0.1 | 44.4 ± 0.3 | 44.7 ± 0.5 |
|  | **L415W** | 44.2 ± 0.6 | 49.1 ± 0.5 | 48.3 ± 0.5 | 47.4 ± 0.6 |

**Supplementary Table 2: Dissociation constants (*K_d_*) of BmrA NBD mutants for ADP derived from chemical shift perturbation assays.**

| **Construct** | ***K_d_* _ADP_ (µM)** |
| --- | --- |
| BmrA NBD WT | 214.1 ± 42.9 |
| BmrA NBD K380A | 14416 ± 3704 |
| BmrA NBD R389K | 220.8 ± 105.0 |
| BmrA NBD R389M | 2887.8 ± 1461.3 |
| BmrA NBD W413F | 220.7 ± 43.3 |
| BmrA NBD R414A | 575.1 ±43.5 |
| BmrA NBD R414K | 265.6 ± 96.5 |

**Supplementary Table 3: Effect of hinge mutants on the thermal stability of full-length BmrA.** Melting temperatures (*T_m_*) of protein in DDM/Cholate detergent micelles were determined using nanoDSF (R414X, W413X) or the fluorescent GloMelt™assay (R398X and ^19^F-5Trp-labeled samples) in the apo state (nucleotide free) and in presence of MgADP*V_i_ to stabilize the nucleotide bound state. Results are shown as mean ± standard deviation from two biological replicates measured three times or, in the case of the ^19^F-labeled samples, from one biological replicate measured three times.

| **Full-length BmrA construct** | ***Tm* _apo_ (ºC)** | ***Tm* _MgADP*Vi_ (ºC)** |
| --- | --- | --- |
| **WT** | 43.6 ± 1.0 | 51.2 ± 0.3 |
| **K380A** | 44.0 ± 0.3 | 44.9 ± 0.1 |
| **R389A** | 38.7 ± 0.3 | 43.9 ± 3.0 |
| **R389E** | 39.5 ± 1.0 | 43.6 ± 0.4 |
| **R389K** | 40.1 ± 0.1 | 43.7 ± 0.8 |
| **R389M** | 38.5 ± 1.2 | 48.1 ± 1.7 |
| **W413A** | 39.6 ± 1.7 | 42.2 ± 1 |
| **W413L** | 34.7 ± 0.4 | 49.8 ± 0.5 |
| **W413F** | 36.5 ± 0.3 | 52.0 ± 0.4 |
| **W413Y** | 37.1 ± 0.1 | 50.7 ± 0.6 |
| **R414A** | 37.4 ± 0.0 | 38.0 ± 0.0 |
| **R414K** | 39.3 ± 0.1 | 40.6 ± 0.1 |
| **^19^F–5W WT** | 42.6 ± 1.7 | 56.3 ± 2.4 |
| **^19^F–5W W104F** | 37.2 ± 0.8 | 57.5 ± 0.3 |
| **^19^F–5W W164F** | 43.0 ± 1.8 | 61.5 ± 1.2 |
| **^19^F–5W W413Y** | 45.9 ± 2.2 | 58.4 ± 1.1 |

**Supplementary Table 4: HDX-MS report summary.**

| **Data** | **BmrA WT (Apo)** | **BmrA WT (ADP*V_i_)** | **BmrA WT (Doxo)** | **BmrA W413F (Apo)** | **BmrA W413F (ADP*V_i_)** | **BmrA W413F (Doxo)** |
| --- | --- | --- | --- | --- | --- | --- |
| HDX reaction details | 20-fold dilution in labeling buffer^1^ (95% D2O, pD 8, 20°C) | | | | | |
| HDX quench details | Quench^2^ at 0°C, final pH=2.5 | | | | | |
| HDX time course | 0, 2, 5, 15 and 30 min | | | | | |
| HDX controls | Pepsin washes performed during each run / Blank injected between each time point | | | | | |
| Back exchange | No correction for back-exchange using fully deuterated samples conducted. | | | | | |
| Number of peptides | 141 | 136 | 137 | 155 | 155 | 155 |
| Sequence coverage | 96.3% | 93% | 96.3% | 90.6% | 90.6% | 90.6% |
| Average peptide length/redundancy | 10.70 / 2.63 | 10.45 / 2.56 | 10.64 / 2.54 | 11.48 / 3.30 | 11.48 / 3.30 | 11.48 / 3.30 |
| Replicates (technical) | 2 | | | 3 | | |
| Repeatability (average SD) | 0.035 | 0.0357 | 0.0349 | 0.0553 | 0.0598 | 0.0536 |
| Significant differences in HDX | Peptide significance^5^ statistical tests using a *p-value* threshold of 0.05 | | | | | |

^1^Labeling buffer additionally contained 10 mM ATP, 10 mM MgCl_2_, 1 mM Vi for (ADP*V_i_) or 100 μM doxorubicin for (100µM-Doxo) conditions. Prior to labeling, the samples were incubated with the respective ligands for 15 min at 20 °C.

^2^Buffer composition: 0.5 M glycine, 8 M guanidine-HCl pH 2.2, 0.035% DDM, 0.03% sodium cholate. Quench was followed by an off-line delipidation using 200 μg of activated zirconium magnetic beads.

**Supplementary Table 5: Comparison of HDX results for BmrA WT and W413F mutant.**

Ninety-five shared peptides were identified between the WT and the W413F mutant (80% sequence coverage, see Supplementary Figure 9). The relative differences in uptake (∆uptake) between W413F and WT for identical peptides, normalized by the maximal uptake of deuteration (expressed as a percentage), are shown. Values were taken at 15 min deuteration in the apo state (see Supplementary Figure 9 for the corresponding uptake curves). Peptides with a similar level of deuteration (< 5% difference) between the W413F mutant and the WT protein are indicated. Peptides where the deuteration levels are between ≥ 5% and <10% higher in the mutant compared to the WT are colored in pink. Peptides with a difference of ≥ 10% for the mutant are colored in red. Peptides that showed higher deuteration in the WT are colored in blue.

The outward facing (OF) conformation was obtained by trapping the transporter with ADP*V_i_. The protection from deuterium uptake in the OF conformation is indicated in the last column for the WT and the W413F mutant, ranging from no protection (-), to high protection (+++). Examples of the most significant peptides, which displayed similar deuteration levels for WT and W413F mutant (apo state) but showed a significant difference in the ADP*Vi state, are highlighted in green. These 6 peptides were used to generate the Figure 4.

| **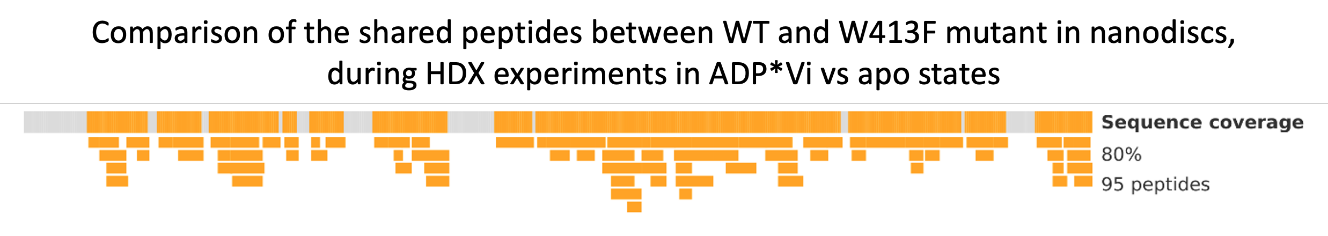** | | | | | | | |
| --- | --- | --- | --- | --- | --- | --- | --- |
| **Peptide** | | **Sequence** | **Max. Uptake** | **∆uptake (W413F -WT) /max uptake (in %) in the apo state** | **Low level of deuteration**  **(< 5%)** | **Protection HDX in OF (ADP*V_i_)** | |
| **Start** | **End** |  |  |  |  | **WT** | **W413F** |
| 36 | 41 | SVVTTL | 5 | Similar level | Yes |  |  |
| 42 | 52 | VSLLIPLLTKQ | 9 | Similar level | Yes |  |  |
| 42 | 56 | VSLLIPLLTKQLVDG | 13 | Similar level | Yes |  |  |
| 46 | 56 | IPLLTKQLVDG | 9 | Similar level | Yes |  |  |
| 46 | 57 | IPLLTKQLVDGF | 10 | Similar level | Yes |  |  |
| 57 | 69 | FSMSNLSGTQIGL | 12 | 10.2 |  | - | - |
| 63 | 69 | SGTQIGL | 6 | 10.7 |  | - | - |
| 75 | 82 | FVQAGLSA | 7 | Similar level | Yes |  |  |
| 83 | 88 | YATYAL | 5 | Similar level | Yes |  |  |
| 86 | 99 | YALNYNGQKIISGL | 13 | 6.7 |  | - | - |
| 89 | 99 | NYNGQKIISGL | 10 | 6.2 |  | - | - |
| 104 | 114 | WKKLIKLPVSY | 9 | 14 |  | + | +/- |
| 108 | 114 | IKLPVSY | 5 | 11.8 |  | ++ | + |
| 108 | 115 | IKLPVSYF | 6 | 12.8 |  | ++ | + |
| 115 | 130 | FDTNASGETVSRVTND | 15 | Similar level |  | + | +/- |
| 115 | 132 | FDTNASGETVSRVTNDTM | 17 | Similar level |  | + | +/- |
| 115 | 133 | FDTNASGETVSRVTNDTMV | 18 | Similar level |  | + | +/- |
| 116 | 132 | DTNASGETVSRVTNDTM | 16 | Similar level |  | + | +/- |
| 133 | 142 | VVKELITTHI | 9 | 23.6 |  | +/- | + |
| 145 | 152 | FITGIISV | 7 | Similar level | Yes |  |  |
| 146 | 152 | ITGIISV | 6 | Similar level | Yes |  |  |
| 160 | 164 | FIMNW | 4 | Similar level | Yes |  |  |
| 160 | 168 | FIMNWKLTL | 8 | Similar level | Yes |  |  |
| 168 | 178 | LLVLVVVPLAA | 9 | 7.3 |  | +/- | - |
| 195 | 202 | TQDETARF | 7 | 8.1 |  | ++ | + |
| 203 | 210 | TGLLNQIL | 7 | 6.8 |  | +++ | + |
| 206 | 210 | LNQIL | 4 | 6.2 |  | +++ | + |
| 207 | 215 | NQILPEIRL | 7 | Similar level |  | +++ | + |
| 210 | 214 | LPEIR | 3 | Similar level |  | +++ | + |
| **Supplementary Table 5 (continued)** | | | | | | | |
| 216 | 225 | VKASNAEDVE | 9 | Similar level |  | ++ | +/- |
| 216 | 236 | VKASNAEDVEYGRGKMGISSL | 20 | Similar level |  | ++ | + |
| 223 | 236 | DVEYGRGKMGISSL | 13 | Similar level |  | ++ | + |
| 224 | 236 | VEYGRGKMGISSL | 12 | Similar level |  | ++ | + |
| 263 | 283 | VAVIGYGGMQVSSGELTAGAL | 20 | Similar level | Yes |  |  |
| 286 | 290 | FILYL | 4 | Similar level | Yes |  |  |
| 291 | 303 | FQIIMPMGQITTF | 11 | Similar level | Yes |  |  |
| 293 | 303 | IIMPMGQITTF | 9 | Similar level | Yes |  |  |
| 304 | 317 | FTQLQKSIGATERM | 13 | 8.3 |  | + | +/- |
| 308 | 317 | QKSIGATERM | 9 | 7.8 |  | +/- | - |
| 318 | 323 | IEILAE | 5 | 5.2 |  | +/- | - |
| 322 | 339 | AEEEEDTVTGKQIENAHL | 17 | 12.4 |  | +/- | +/- |
| 322 | 343 | AEEEEDTVTGKQIENAHLPIQL | 20 | 13.3 |  | +/- | +/- |
| 325 | 343 | EEDTVTGKQIENAHLPIQL | 17 | 11.6 |  | +/- | +/- |
| 327 | 339 | DTVTGKQIENAHL | 12 | 6.3 |  | + | - |
| 327 | 343 | DTVTGKQIENAHLPIQL | 15 | 11.1 |  | +/- | +/- |
| 336 | 343 | NAHLPIQL | 6 | 27.9 |  | - | + |
| 344 | 357 | DRVSFGYKPDQLIL | 12 | 10.3 |  | + | + |
| 344 | 355 | DRVSFGYKPDQL | 10 | 5.5 |  | - | +/- |
| 344 | 348 | DRVSF | 4 | 29.9 |  | - | + |
| 349 | 355 | GYKPDQL | 5 | Similar level |  | +/- | +/- |
| 349 | 357 | GYKPDQLIL | 7 | 7.2 |  | + | + |
| 356 | 362 | ILKEVSA | 6 | 8.7 |  | +/- | + |
| 362 | 371 | AVIEAGKVTA | 9 | 18 |  | - | + |
| 362 | 383 | AVIEAGKVTAIVGPSGGGKTTL | 20 | 5.8 |  | + | + |
| 363 | 371 | VIEAGKVTA | 8 | 16 |  | - | + |
| 363 | 383 | VIEAGKVTAIVGPSGGGKTTL | 19 | Similar level |  | ++ | + |
| 365 | 371 | EAGKVTA | 6 | 10.8 |  | - | +/- |
| 372 | 383 | IVGPSGGGKTTL | 10 | Similar level |  | ++ | + |
| 384 | 397 | FKLLERFYSPTAGT | 12 | 9.9 |  | + | + |
| 384 | 390 | FKLLERF | 6 | 16.7 |  | + | + |
| 391 | 397 | YSPTAGT | 5 | Similar level |  | - | - |
| 391 | 407 | YSPTAGTIRLGDEPVDT | 14 | 14 |  | +/- | + |
| 398 | 407 | IRLGDEPVDT | 8 | 13.3 |  | - | +/- |
| 408 | 413 | YSLESF | 5 | Similar level |  | + | + |
| 413 | 427 | W/FREHIGYVSQESPLM | 13 | Similar level |  | + | + |
| 413 | 430 | FREHIGYVSQESPLMSGT | 16 | Similar level |  | + | + |
| 420 | 430 | VSQESPLMSGT | 9 | Similar level |  | + | + |
| 420 | 433 | VSQESPLMSGTIRE | 12 | Similar level |  | + | + |
| 434 | 439 | NICYGL | 5 | Similar level |  | +/- | +/- |
| 438 | 447 | GLERDVTDAE | 9 | Similar level |  | - | - |
| 440 | 447 | ERDVTDAE | 7 | Similar level |  | - | - |
| 447 | 455 | EIEKAAEMA | 8 | -7.3 |  | +/- | - |
| 460 | 468 | FIKELPNQF | 7 | Similar level |  | - | - |
| 461 | 468 | IKELPNQF | 6 | Similar level |  | - | - |
| 469 | 478 | DTEVGERGIM | 9 | Similar level |  | ++ | + |
| 479 | 490 | LSGGQRQRIAIA | 11 | 9.8 |  | ++ | + |
| 491 | 500 | RALLRNPSIL | 8 | 17.9 |  | - | +/- |
| 493 | 500 | LLRNPSIL | 6 | 16.1 |  | - | +/- |
| 494 | 500 | LRNPSIL | 5 | 16.9 |  | - | +/- |
| 501 | 509 | MLDEATSSL | 8 | Similar level |  | ++ | + |
| 502 | 509 | LDEATSSL | 7 | Similar level |  | ++ | + |
| 510 | 522 | DSQSEKSVQQALE | 12 | Similar level |  | ++ | + |
| 525 | 539 | MEGRTTIVIAHRLST | 14 | Similar level |  | - | - |
| 530 | 539 | TIVIAHRLST | 9 | Similar level |  | +/- | - |
| 540 | 547 | VVDADQLL | 7 | 16.7 |  | - | + |
| 564 | 569 | MASHGL | 5 | Similar level |  | - | +/- |
| 570 | 577 | YRDFAEQQ | 7 | Similar level |  | +/- | +/- |
| 570 | 578 | YRDFAEQQL | 8 | Similar level |  | +/- | +/- |
| 573 | 578 | FAEQQL | 5 | Similar level |  | - | - |
| 573 | 580 | FAEQQLKM | 7 | Similar level |  | - | - |
| 579 | 584 | KMNADL | 5 | -12.9 |  | - | - |
| 581 | 593 | NADLENKAGVDKL | 12 | Similar level |  | - | - |
| 581 | 595 | NADLENKAGVDKLAA | 14 | Similar level |  | - | - |
| 585 | 593 | ENKAGVDKL | 8 | Similar level |  | - | - |
| 585 | 595 | ENKAGVDKLAA | 10 | Similar level |  | - | - |

**Supplementary Table 6: Sequences of primers used to obtain hinge and tryptophan mutants**

| **Primer name** | **DNA Sequence** |
| --- | --- |
| BmrA NBD K380A forward | 5’ – GAGCGGTGGCGGTGCGACCACCCTGTTC – 3’ |
| BmrA NBD K380A reverse | 5’ – GTTTGAACAGGGTGGTCGCACCGCCACCGC – 3’ |
| BmrA NBD R389A forward | 5’ – CTGTTCAAACTGCTGGAGGCGTTTTATAGCCCGAC – 3’ |
| BmrA NBD R389A reverse | 5’ – GCGGTCGGGCTATAAAACGCCTCCAGCAG - 3’ |
| BmrA NBD R389E forward | 5’ – CCTGTTCAAACTGCTGGAGGAATTTTATAGCC – 3’ |
| BmrA NBD R389E reverse | 5’ – GTCGGGCTATAAAATTCCTCCAGCAGTTTG – 3’ |
| BmrA NBD R389K forward | 5’ – CCTGTTCAAACTGCTGGAGAAATTTTATAGCC – 3’ |
| BmrA NBD R389K reverse | 5’ – GTCGGGCTATAAAATTTCTCCAGCAGTTTG – 3’ |
| BmrA NBD R389M forward | 5’ – CCTGTTCAAACTGCTGGAGATGTTTTATAGCC – 3’ |
| BmrA NBD R389M reverse | 5’ – GTCGGGCTATAAAACATCTCCAGCAGTTTG – 3’ |
| BmrA NBD W413A forward | 5’ – CTACAGCCTGGAGAGCGCGCGTGAACAC – 3’ |
| BmrA NBD W413A reverse | 5’ – CATAACCAATGTGTTCACGCGCGCTCTCCAG – 3’ |
| BmrA NBD W413L forward | 5’ – GATACCTACAGCCTGGAGAGCCTGCGTGAACAC – 3’ |
| BmrA NBD W413L reverse | 5’ – GTATTGGTTACACTTCACGCAGGCTCTCCAGGCTG – 3’ |
| BmrA NBD W413F forward | 5’ – GATACCTACAGCCTGGAGAGCTTTCGTGAACAC – 3’ |
| BmrA NBD W413F reverse | 5’ – CATAACCAATGTGTTCACGAAAGCTCTCCAGGCTG – 3’ |
| BmrA NBD W413Y forward | 5’ – CCTACAGCCTGGAGAGCTATCGTGAAC – 3’ |
| BmrA NBD W413Y reverse | 5’ – CATAACCAATGTGTTCACGATAGCTCTCCAG – 3’ |
| BmrA NBD R414A forward | 5’ – CTACAGCCTGGAGAGCTGGGCGGAACACATTG – 3’ |
| BmrA NBD R414A reverse | 5’ – CACCATAACCAATGTGTTCCGCCCAGCTCTC – 3’ |
| BmrA NBD R414K forward | 5’ – CTACAGCCTGGAGAGCTGGAAGGAACACATTG – 3’ |
| BmrA NBD R414K reverse | 5’ – CACCATAACCAATGTGTTCCTTCCAGCTCTC – 3’ |
| LmrA NBD W421A forward | 5’ – GATAGCGTGAGCCTGGAGAACGCGCGTAGCCAG – 3’ |
| LmrA NBD W421A reverse | 5’ – CTAACGAAGCCGATCTGGCTACGCGCGTTCTCCAG – 3’ |
| LmrA NBD R397A forward | 5’ – GCACCATTTTCAGCCTGCTGGAGGCGTTTTATCAG – 3’ |
| LmrA NBD R397A reverse | 5’ – CGCGGTCGGCTGATAAAACGCCTCCAGCAG – 3’ |
| MsbA NBD R391A forward | 5’ – CCATCGCCAGCCTGATCACGGCTTTTTACG – 3’ |
| MsbA NBD R391A reverse | 5’ – GCCTTCATCAATATCGTAAAAAGCCGTGATC – 3’ |
| MsbA NBD L415A forward | 5’ – GAGTATACCCTGGCGTCGGCACGTAAC – 3’ |
| MsbA NBD L415A reverse | 5’ – CAGAGCAACCTGGTTACGTGCCGACGC – 3’ |
| MsbA NBD L415W forward | 5’ – CGCGAGTATACCCTGGCGTCGTGGCGTAACC – 3’ |
| MsbA NBD L415W reverse | 5’ – CACCAGAGCAACCTGGTTACGCCACGACGC – 3’ |

**Supplementary Table 6 (continued)**

| BmrA NBD C436S forward | 5’ – CCATTCGTGAGAACATCAGCTACGGTCTGG – 3’ |
| --- | --- |
| BmrA NBD C436S reverse | 5’ – CACGTTCCAGACCGTAGCTGATGTTCTC – 3’ |
| BmrA NBD N459W forward | 5’ – CGGAGATGGCGTATGCGCTGTGGTTCATTAAAG – 3’ |
| BmrA NBD N459W reverse | 5’ – GTTCGGCAGTTCTTTAATGAACCACAGCGCATAC – 3’ |
| BmrA NBD S516C forward | 5’ – CTGGATAGCCAGAGCGAAAAGTGCGTTCAGCAAG – 3’ |
| BmrA NBD S516C reverse | 5’ – CTTCCAGCGCTTGCTGAACGCACTTTTCGCTC – 3’ |
| BmrA full-length K380A forward | 5’ – GCGGCGGGGGAGCGACGACGCTGTTTAAG – 3’ |
| BmrA full-length K380A reverse | 5’ – CTTAAACAGCGTCGTCGCTCCCCCGCCG – 3’ |
| BmrA full-length R389A forward | 5’ – CGCTGTTTAAGCTGCTTGAAGCGTTTTATTCTCCG – 3’ |
| BmrA full-length R389A reverse | 5’ – CTGCAGTCGGAGAATAAAACGCTTCAAGCAGC – 3’ |
| BmrA full-length R389E forward | 5’ – CGCTGTTTAAGCTGCTTGAAGAATTTTATTCTCCG – 3’ |
| BmrA full-length R389E reverse | 5’ – CTGCAGTCGGAGAATAAAATTCTTCAAGCAGC – 3’ |
| BmrA full-length R389K forward | 5’ – CGCTGTTTAAGCTGCTTGAAAAATTTTATTCTCCG – 3’ |
| BmrA full-length R389K reverse | 5’ – CTGCAGTCGGAGAATAAAATTTTTCAAGCAGC – 3’ |
| BmrA full-length R389M forward | 5’ – CGCTGTTTAAGCTGCTTGAAATGTTTTATTCTCCG – 3’ |
| BmrA full-length R389M reverse | 5’ – CTGCAGTCGGAGAATAAAACATTTCAAGCAGC – 3’ |
| BmrA full-length W413A forward* | 5’ –ACCCGATATGCTCCCTCGCCGACTCGAGCGAGTAAGTAT– 3’ |
| BmrA full-length W413L forward* | 5’ –ACCCGATATGCTCCCTCAACGACTCGAGCGAGTAAGTAT– 3’ |
| BmrA full-length W413F forward* | 5’ –ACCCGATATGCTCCCTAAACGACTCGAGCGAGTAAGTAT– 3’ |
| BmrA full-length W413Y forward* | 5’ –ACCCGATATGCTCCCTATACGACTCGAGCGAGTAAGTAT– 3’ |
| BmrA full-length R414A forward | 5’ –ACTCGCTTGAATCGTGGGCGGAGCATATCGGGTATG– 3’ |
| BmrA full-length R414A reverse | 5’ –CATACCCGATATGCTCCGCCCACGATTCAAGCGAGT– 3’ |
| BmrA full-length R414K forward | 5’ –CTCGCTTGAATCGTGGAAGGAGCATATCGGGTATG– 3’ |
| BmrA full-length R414K reverse | 5’ –CATACCCGATATGCTCCTTCCACGATTCAAGCGAG– 3’ |
| BmrA full-length W104F forward | 5’ –CCGGGCTGCGGGAGTTATTATTTAAGAAATT– 3’ |
| BmrA full-length W104F reverse | 5’ –GGCAGCTTAATTAATTTCTTAAATAATAACTCC– 3’ |
| BmrA full-length W164F forward | 5’ –CAATCTTGTTTATTATGAACTTTAAGCTGACAC– 3’ |
| BmrA full-length W164F reverse | 5’ –CTAACACAAGCAGTGTCAGCTTAAAGTTCATAAT– 3’ |

* Only one primer was used following the same cloning technique as in Orelle et al., 2003^6^.

**Supplementary References**

1. Di Cesare, M. *et al.* The transport activity of the multidrug ABC transporter BmrA does not require a wide separation of the nucleotide-binding domains. *Journal of Biological Chemistry* 105546 (2024) doi:10.1016/j.jbc.2023.105546.

2. Chaptal, V. *et al.* Substrate-bound and substrate-free outward-facing structures of a multidrug ABC exporter. *Sci Adv* **8**, (2022).

3. Lyu, J. *et al.* Structural basis for lipid and copper regulation of the ABC transporter MsbA. *Nat Commun* **13**, 7291 (2022).

4. Pérez Carrillo, V. H., Rose-Sperling, D., Tran, M. A., Wiedemann, C. & Hellmich, U. A. Backbone NMR assignment of the nucleotide binding domain of the Bacillus subtilis ABC multidrug transporter BmrA in the post-hydrolysis state. *Biomol NMR Assign* **16**, 81–86 (2022).

5. Lau, A. M., Claesen, J., Hansen, K. & Politis, A. Deuteros 2.0: peptide-level significance testing of data from hydrogen deuterium exchange mass spectrometry. *Bioinformatics* **37**, 270–272 (2021).

6. Orelle, C., Dalmas, O., Gros, P., Di Pietro, A. & Jault, J.-M. The Conserved Glutamate Residue Adjacent to the Walker-B Motif Is the Catalytic Base for ATP Hydrolysis in the ATP-binding Cassette Transporter BmrA. *Journal of Biological Chemistry* **278**, 47002–47008 (2003).
