# Supplementary material for: Bidirectional communication between nucleotide and substrate binding sites in a type IV multidrug ABC transporter
Source: Nat Commun. 2025 Nov 11;16:9921. doi: 10.1038/s41467-025-65037-y (PMC12606247; doi:10.1038/s41467-025-65037-y)
Supplement: Supplementary file 2 — Reporting Summary [file 41467_2025_65037_MOESM2_ESM.pdf]

nature portfolio

Corresponding author(s): U Hellmich, JM Jault, C Orelle

Last updated by author(s): Aug 22, 2025

Reporting Summary

Nature Portfolio wishes to improve the reproducibility of the work that we publish. This form provides structure for consistency and transparency in reporting. For further information on Nature Portfolio policies, see our Editorial Policies and the Editorial Policy Checklist.

Statistics

For all statistical analyses, confirm that the following items are present in the figure legend, table legend, main text, or Methods section.

n/a	Confirmed	
☐	☒	The exact sample size (*n*) for each experimental group/condition, given as a discrete number and unit of measurement
☐	☒	A statement on whether measurements were taken from distinct samples or whether the same sample was measured repeatedly
☐	☒	The statistical test(s) used AND whether they are one- or two-sided<br>*Only common tests should be described solely by name; describe more complex techniques in the Methods section.*
☒	☐	A description of all covariates tested
☒	☐	A description of any assumptions or corrections, such as tests of normality and adjustment for multiple comparisons
☐	☒	A full description of the statistical parameters including central tendency (e.g. means) or other basic estimates (e.g. regression coefficient) AND variation (e.g. standard deviation) or associated estimates of uncertainty (e.g. confidence intervals)
☒	☐	For null hypothesis testing, the test statistic (e.g. *F*, *t*, *r*) with confidence intervals, effect sizes, degrees of freedom and *P* value noted<br>*Give P values as exact values whenever suitable.*
☒	☐	For Bayesian analysis, information on the choice of priors and Markov chain Monte Carlo settings
☒	☐	For hierarchical and complex designs, identification of the appropriate level for tests and full reporting of outcomes
☒	☐	Estimates of effect sizes (e.g. Cohen's *d*, Pearson's *r*), indicating how they were calculated

Our web collection on statistics for biologists contains articles on many of the points above.

Software and code

Policy information about availability of computer code

Data collection	Sequence alignment was carried out with Clustal Omega (https://www.ebi.ac.uk/jdispatcher/msa/clustalo) and sequence logos created using WebLogo 3 (https://weblogo.threeplusone.com/).<br>FCS data were recorded using the ALV-6010/160 software (ALV, Langen Germany)<br>NMR data were collected on a 600MHz spectrometer using Bruker TopSpin 4.0.8.<br>HDX-MS data were collected with MassLynx 4.1 and HDxDirector 1.0.3.9.
Data analysis	NMR spectra were analyzed with Bruker TopSpin 4.0.8, CARA and CCP NMR.<br>FCS data were analyzed using the commercial software packages Origin 7 and OriginPro 2016G from OriginLab.<br>HDX-MS data were analyzed with PLGS™ software (ProteinLynx Global SERVER 3.0.2 from Waters™), DynamX 3.0 software (Waters™), and Deuteros 2.0 software.

For manuscripts utilizing custom algorithms or software that are central to the research but not yet described in published literature, software must be made available to editors and reviewers. We strongly encourage code deposition in a community repository (e.g. GitHub). See the Nature Portfolio guidelines for submitting code & software for further information.

Data

Policy information about availability of data

All manuscripts must include a data availability statement. This statement should provide the following information, where applicable:

- Accession codes, unique identifiers, or web links for publicly available datasets
- A description of any restrictions on data availability
- For clinical datasets or third party data, please ensure that the statement adheres to our policy

The backbone NMR assignments of L. lactis LmrA, B. subtilis BmrA and E. coli MsbA have been deposited to the BMRB under the accession numbers 51156, 17660, and 52626, respectively. The mass spectrometry data have been deposited to the ProteomeXchange Consortium via the PRIDE partner repository with the dataset identifier PXD058013.

Research involving human participants, their data, or biological material

Policy information about studies with human participants or human data. See also policy information about sex, gender (identity/presentation), and sexual orientation and race, ethnicity and racism.

Reporting on sex and gender	Not applicable to this study
Reporting on race, ethnicity, or other socially relevant groupings	Not applicable to this study
Population characteristics	Not applicable to this study
Recruitment	Not applicable to this study
Ethics oversight	Not applicable to this study

Note that full information on the approval of the study protocol must also be provided in the manuscript.

Field-specific reporting

Please select the one below that is the best fit for your research. If you are not sure, read the appropriate sections before making your selection.

☒ Life sciences ☐ Behavioural & social sciences ☐ Ecological, evolutionary & environmental sciences

For a reference copy of the document with all sections, see nature.com/documents/nr-reporting-summary-flat.pdf

Life sciences study design

All studies must disclose on these points even when the disclosure is negative.

Sample size	Sample sizes are reported in the material and methods section and follow the respective standars on the field.
Data exclusions	No data exclusions were made.
Replication	Biophysical measurements were carried out at least in biological triplicates or technical triplicates. All replicates were included in the statistical analyses. All experiments were reproducible.
Randomization	Not applicable to this study.
Blinding	Not applicable to this study.

Reporting for specific materials, systems and methods

We require information from authors about some types of materials, experimental systems and methods used in many studies. Here, indicate whether each material, system or method listed is relevant to your study. If you are not sure if a list item applies to your research, read the appropriate section before selecting a response.

Materials & experimental systems

n/a	Involved in the study	
☒	☐	Antibodies
☒	☐	Eukaryotic cell lines
☒	☐	Palaeontology and archaeology
☒	☐	Animals and other organisms
☒	☐	Clinical data
☒	☐	Dual use research of concern
☒	☐	Plants

Methods

n/a	Involved in the study	
☒	☐	ChIP-seq
☒	☐	Flow cytometry
☒	☐	MRI-based neuroimaging

Plants

Seed stocks	Not applicable to this study
Novel plant genotypes	Not applicable to this study
Authentication	Not applicable to this study
